# Molecular Mechanism Operating in Animal Models of Neurogenic Detrusor Overactivity: A Systematic Review Focusing on Bladder Dysfunction of Neurogenic Origin

**DOI:** 10.3390/ijms24043273

**Published:** 2023-02-07

**Authors:** Ana Ferreira, Diogo Nascimento, Célia Duarte Cruz

**Affiliations:** 1 Experimental Biology Unit, Department of Biomedicine, Faculty of Medicine of Porto, University of Porto, 4200-319 Porto, Portugal; 2 Instituto de Investigação e Inovação em Saúde-i3S and IBMC, Universidade do Porto, 4200-319 Porto, Portugal

**Keywords:** animal models, lower urinary tract dysfunction, neurogenic bladder, neurogenic detrusor overactivity, detrusor sphincter dyssynergia

## Abstract

Neurogenic detrusor overactivity (NDO) is a severe lower urinary tract disorder, characterized by urinary urgency, retention, and incontinence, as a result of a neurologic lesion that results in damage in neuronal pathways controlling micturition. The purpose of this review is to provide a comprehensive framework of the currently used animal models for the investigation of this disorder, focusing on the molecular mechanisms of NDO. An electronic search was performed with PubMed and Scopus for literature describing animal models of NDO used in the last 10 years. The search retrieved 648 articles, of which reviews and non-original articles were excluded. After careful selection, 51 studies were included for analysis. Spinal cord injury (SCI) was the most frequently used model to study NDO, followed by animal models of neurodegenerative disorders, meningomyelocele, and stroke. Rats were the most commonly used animal, particularly females. Most studies evaluated bladder function through urodynamic methods, with awake cystometry being particularly preferred. Several molecular mechanisms have been identified, including changes in inflammatory processes, regulation of cell survival, and neuronal receptors. In the NDO bladder, inflammatory markers, apoptosis-related factors, and ischemia- and fibrosis-related molecules were found to be upregulated. Purinergic, cholinergic, and adrenergic receptors were downregulated, as most neuronal markers. In neuronal tissue, neurotrophic factors, apoptosis-related factors, and ischemia-associated molecules are increased, as well as markers of microglial and astrocytes at lesion sites. Animal models of NDO have been crucial for understanding the pathophysiology of lower urinary tract (LUT) dysfunction. Despite the heterogeneity of animal models for NDO onset, most studies rely on traumatic SCI models rather than other NDO-driven pathologies, which may result in some issues when translating pre-clinical observations to clinical settings other than SCI.

## 1. Introduction 

The lower urinary tract (LUT), comprising the urinary bladder and urethra, is responsible for the storage and periodic elimination of urine. Micturition relies on the synchronized activity of the bladder and the urethral sphincter, the functional muscular unit that controls urine flux [1,2]. Thus, the expulsive contractions of the detrusor muscle are tightly coordinated with relaxation of the urethral sphincter, to ensure efficient urine removal. Normal LUT function relies on complex networks involving neurons operating in an on–off switch-like manner and located in the peripheral ganglia, spinal cord, and supraspinal centers [1,3]. These neuronal circuits are established and matured during infancy when voluntary control over micturition is learned. Activation of these circuits allows conscious and voluntary switching from storage to voiding, influenced by the perceived state of bladder fullness and assessment of the social appropriateness [1]. Therefore, the complexity of neuronal LUT control is such, that it comes as no surprise that voluntary control over micturition is easily jeopardized in neurologic conditions affecting the central nervous system (CNS), including spinal cord injury (SCI), stroke, and progressive neurodegenerative disorders, such as Parkinson’s disease (PD) or multiple sclerosis (MS) [4,5,6].

The most common urinary dysfunction arising from central neurologic disease is neurogenic detrusor overactivity (NDO), defined by the International Continence Society as “Involuntary detrusor muscle contractions that occur near or at the maximum cystometric capacity, in the setting of a clinically relevant neurologic disease. These contractions generally cannot be suppressed, resulting in urinary incontinence or even reflex bladder emptying (reflex voiding).” [7]. The region where the lesions occur is pivotal for its clinical manifestations. When NDO arises from damages in suprapontine areas (e. g. stroke, PD), symptoms reflect the blockade of tonic inhibition of the pontine micturition center, resulting from damages to supraspinal micturition pathways. These damages are mostly associated with storage symptoms, particularly manifested as detrusor overactivity, as a result of bladder outlet obstruction and urethral sphincter dysfunction [8,9]. 

If the injury occurs due to damages in the suprasacral spinal cord (e.g., SCI), this triggers the emergence of alternative micturition pathways, totally located at the lumbosacral spinal cord, operating in the absence of supraspinal input, and dependent on afferent C fibers [10,11,12]. Unlike what happens after suprapontine lesions, in this case NDO is often concurrent with detrusor sphincter dyssynergia (DSD), resulting in impaired bladder emptying, high residual volumes of urine and frequent episodes of urinary incontinence [5]. There are also cases when the damages may originate from both suprapontine and suprasacral lesions in diseases such as MS, for example. NDO is also likely to occur due to neurogenic inflammation of the bladder, when a physical interruption of brain–bladder circuits is not evident [13,14].

Pharmacological NDO treatment aims to reduce detrusor contractions and promote continence. It is initiated with anticholinergic drugs, with intradetrusor injections of botulinum toxin A remaining the gold standard option for refractory patients [15]. Pharmacological interventions are combined with intermittent catheterization, performed by the patient or the caregiver [16]. NDO courses have increased frequency of urinary tract infections and the risk of kidney deterioration is high [17]. Therefore, health and quality of life of NDO patients is severely compromised. Moreover, available therapies often carry significant side effects, and some may lose efficiency over time. Therefore, a breakthrough in the treatment of NDO is urgently needed. To produce a significant advance in NDO therapies, it is necessary to gain a better understanding of NDO pathophysiology and grasp molecular changes that underly this pathology, including changes in the expression of many receptors, trophic factors, and inflammatory mediators. Animal models have been critical for this, as they offer the possibility to investigate in vivo consequences of NDO, including shifts in urodynamic parameters, and identify changes in gene expression and electrophysiological properties of neurons involved in the control of LUT function [6,11]. Furthermore, by mimicking human pathology, the back translational value of animal models becomes more evident, as they allow the direct testing of new drugs and therapies before advancing research to clinical trials. 

There are many studies using animal models of NDO but published results are diverse and difficult to interpret in an integrative manner. However, to propose truly innovative hypotheses, it is necessary to congregate data and critically review published results, appreciating the wealth of knowledge generated. Therefore, the present systematic review aims to collect and discuss pre-clinical literature focusing on molecular changes associated with NDO of central origin, providing an up-to-date analysis of molecular studies involving animal models to assess NDO published in the last decade. This review seeks to present a holistic view of the current findings in animal NDO models, which is essential to shape future research directions in the field and propel clinical translation findings. 

## 2. Results

The fine molecular mechanisms involved in NDO emergence and maintenance are currently better understood [18], but remain challenging for clinicians and researchers. A fully effective treatment, able to revert urinary dysfunction, remains to be identified and there is ample need to improve symptomatic care for NDO patients and, as a consequence, their quality of life. Current treatment aims primarily to protect the upper urinary tract and, later on, to promote continence. However, treatments are not fully effective, carry bothersome secondary effects, such as cognitive impairment (due to prolonged use of anti-muscarinic drugs) and increased frequency of urinary infections, and are not able to revert loss of control over bladder function [19,20]. Therefore, animal models of NDO are critical to clarify NDO pathophysiology and pinpoint putative therapeutic targets. 

**Figure 1:**
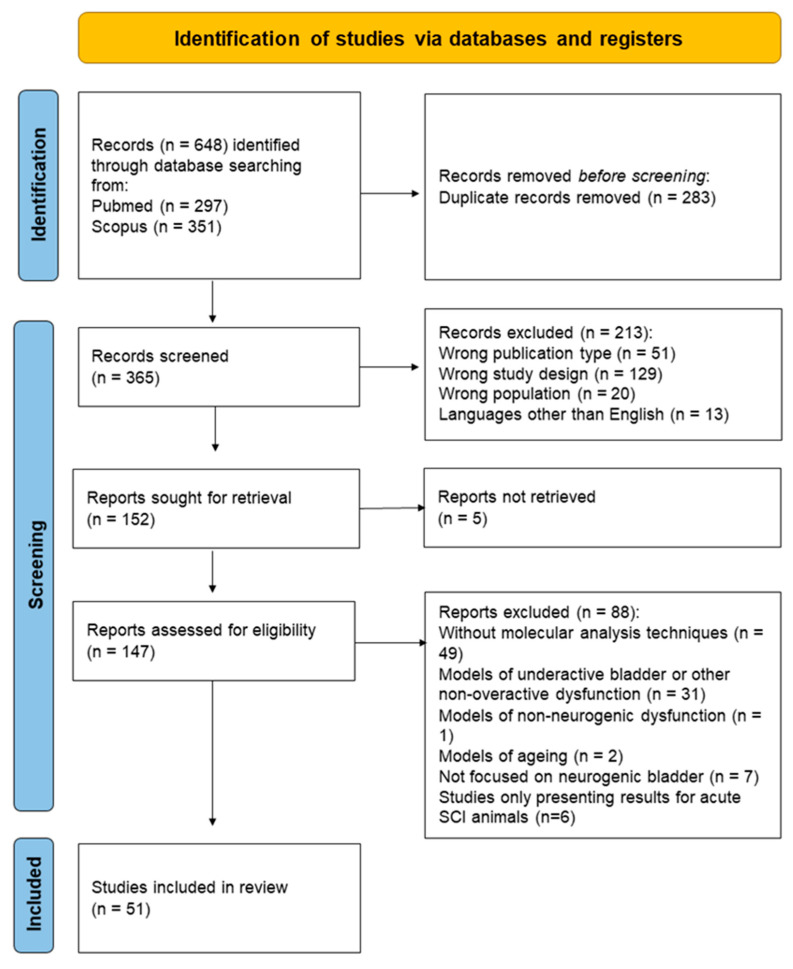
PRISMA flow diagram with the screening process for the present systematic review.

The database search used the key words ((Animal model) OR (rat) OR (mice) OR (rabbit) OR (pig)) AND ((Neurogenic detrusor overactivity) OR (neurogenic bladder)) and identified 648 candidate studies. After the removal of duplicate records, 365 studies were screened by title and abstract. Two hundred and thirteen records were excluded: 129 were unrelated studies, 13 studies were published in languages other than English, and 20 records focused on human patients as their study population. Fifty-one publications were also excluded because they were not journal articles, and included reviews (n = 42), conference papers (n = 2), and comments or editorials (n = 7). From the remaining 152 studies, it was not possible to retrieve 5 publications. The full texts of 147 articles were screened according to the inclusion and exclusion criteria (see below), which resulted in 51 eligible studies for this systematic review. From the 147 screened articles, 2 reports were excluded, as they used aging as an NDO model but failed to indicate neuronal causes for this urinary dysfunction. Finally, 6 publications were further dismissed, as they used spinal cord injury (SCI) as an NDO model but only focused on early-stage SCI, i.e., 14 days after spinal injury, when animals are still in spinal shock [21,22,23] and there are little or no signs of bladder reflex activity [24,25,26,27]. They also failed to produce urodynamic data indicative of NDO within that time frame. The study selection process is depicted in Figure 1, and a summary of the included studies is presented in Table 1. 

### 2.1. Induction Model and Assessment of Bladder Function

**Figure 2:**
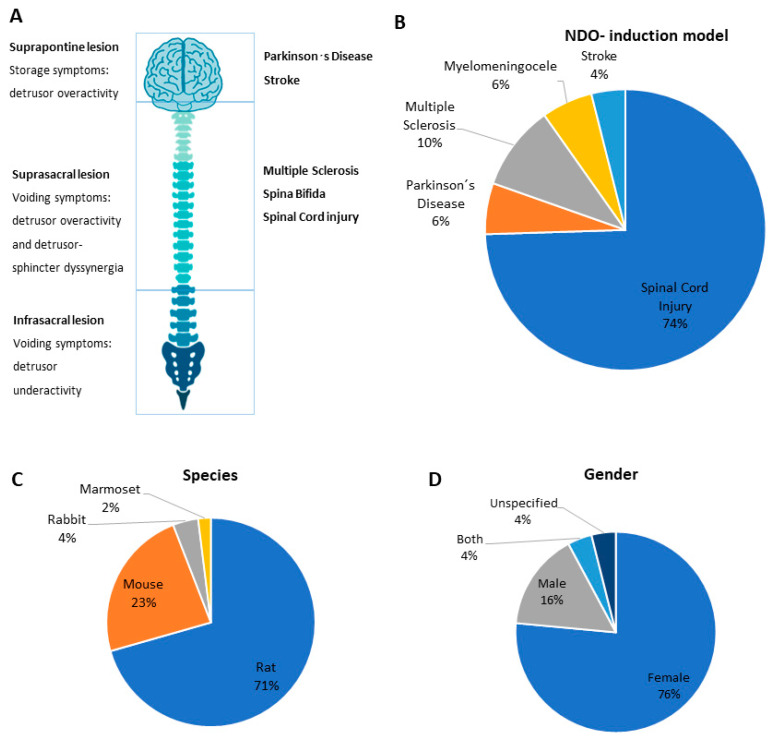
NDO-driven pathologies and animal models to induce them. (**A**) The consequences of central nervous system (CNS) injuries for urinary function are influenced by the level on which they occur: supraspontine lesions are associated with storage symptoms, suprasacral lesions are characterized by voiding symptoms, namely detrusor overactivity and detrusor sphincter dyssynergia, and infrasacral lesions are often associated with detrusor underactivity. (**B**) Animal models to induce neurogenic detrusor overactivity (NDO) are based on supraspontine or suprasacral lesions and include animal models of spinal cord injury (SCI), Parkinson’s disease (PD), multiple sclerosis (MS), myelomeningocele, and stroke. (**C**) The species used to induce NDO models are predominantly rats and mice, with little use of other small mammals, such as rabbits and marmosets. (**D**) Female animals were predominantly used.

**Figure 3:**
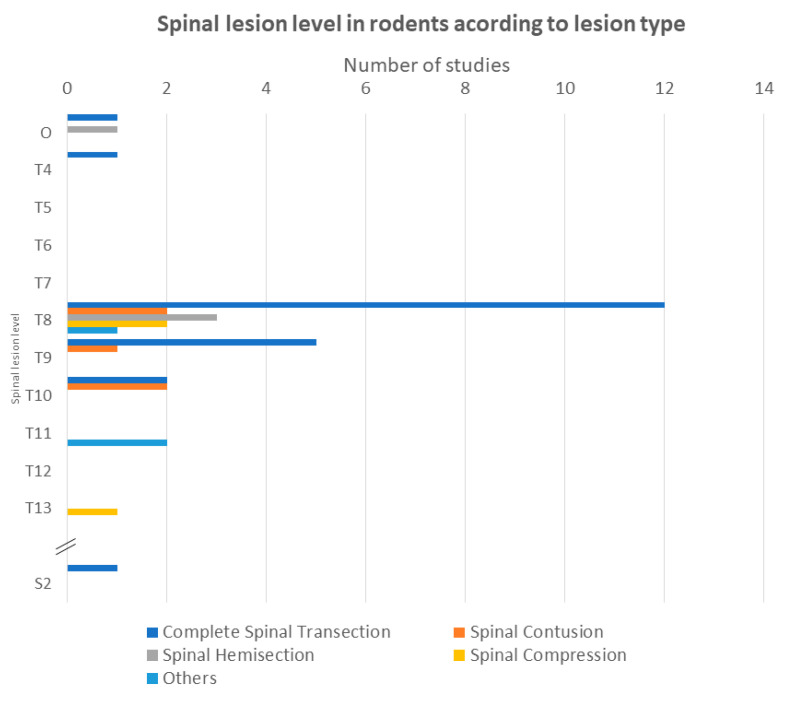
Injured spinal cord levels according to lesion type. O: Other—this category includes one publication which did not specify the spinal lesion level and another study that reported a complex spinal cord injury (involving non-contiguous spinal segments). Since many studies present their spinal lesion level as a combined lesion of two contiguous segments (e.g., T8–T9), we only considered the upper segment in order to graphically represent these data. This graph only represents data reported from rodents (rats and mice) due to their similar vertebral formula. Only two studies used non-rodent animals to induce SCI, and both relied on lagomorphs (rabbits)—which have a distinct vertebral formula.

LUT dysfunction is a common consequence of several neurologic diseases. The level at which they occur may provoke distinct urinary complications (Figure 2A), such as NDO [8]. The vast majority of the analyzed reports, 74%, use spinal cord injury as an NDO model (Figure 2B). From these publications, 59% report complete transection of thoracic segments to induce SCI [26,27,28,29,30,31,32,33,34,35,38,39,43,44,46,48,49,50,55,57,62,63], followed by spinal contusion (13%). The use of spinal hemisections was less frequent (10%) [42,47,52], as were spinal compressions (8%) [32,36,54,56] and other SCI methods (10%) [41,51,53,58] (Figure 3). Regarding the SCI model of NDO, we found that, irrespective of the type of injury, the thoracic level was the preferred level to inflict spinal lesion, with the T8–T9 levels being particularly favored. Only one study used a lesion at a higher level (T4) [48] (Figure 3). 

The second most-used animal models to produce NDO were related to neurodegenerative disorders. MS was reported in 10% of the articles reviewed and it was induced by promoting experimental autoimmune encephalomyelitis (EAE) [66,68,69] and coronavirus-induced encephalitis (CIE) [65,67]. PD was reproduced in 6% of the studies, using pharmacological induction [71,73] or genetic models [64]. Animal models associated with meningomyelocele (6%), which used retinoic acid as the induction model, stroke (4%), using middle cerebral artery occlusion (MCAO) [70] to resemble cerebral ischemia, and cerebral hemorrhage in the hippocampus to reproduce hemorrhagic stroke [72] were less frequent. 

The vast majority of selected studies (71%) performed urodynamic evaluation of the animals to confirm the presence of NDO. Signs of LUT dysfunction were evident, with animals presenting NDO characteristic features: increased micturition frequency; increased number of non-voiding contractions, basal pressure, maximum voiding pressure, and threshold pressure; and high residual volumes. Moreover, decreased voiding volume, maximum flow rate, and voiding efficiency were also referred to. In cases of traumatic models (such as SCI), the setting of the NDO phenotype was preceded by a period of neurogenic shock, with little or no bladder activity, that lasted up to 14 days post-injury. In the remaining pathologies (MS, PD, stroke), NDO symptoms were present immediately after model induction. 

Most of the studies reporting urodynamic recordings used awake cystometry as the recording method (37%) [31,32,33,34,35,38,39,42,46,47,48,52,56,59,63,68,70,71]. Cystometry under anesthesia represented 26% of studies, with 16% using urethane delivered via subcutaneous [26,27,28,37,41,43,51] or intraperitoneal injection [57], 8% using zolotyl [53,58,61,74], and 2% referring the use of chloral hydrate as anesthetic [54]. Finally, 8% of the studies used non-invasive voiding spot assays to evaluate bladder function [64,65,67,68]. Surprisingly, 29% of the studies did not describe any assessment of lower urinary tract function [29,30,36,44,45,49,50,60,62,66,69,73,74,75,76]. More data on changes in bladder function can be found in Table 1.

### 2.2. Animal Species and Sex

Concerning the animal species used in NDO models, rodents were preferred, with 71% of studies using rats. Other animals were less frequently used and included mice (23%), rabbits (4%), and non-human primates (marmosets) (2%) (Figure 1C). The majority of studies used female animals (76%). Males were used in 16% of selected studies, while only 3% used both male and female animals. Curiously, 5% of studies did not specify which sex was used (Figure 1D).

### 2.3. Changes in Bladder Tissue

Many studies presented significant findings regarding gross tissue and cellular morphology of the bladder. Animals with an NDO phenotype presented larger bladder volumes and weights than control animals. Bladder tissue was also more fibrotic in NDO animals, associated with detrusor hypertrophy, features which led to a bladder wall thickness increase. The urothelial layer was usually damaged and disorganized, and inflammatory features were conspicuous in the NDO phenotype—leucocyte infiltration in the lamina propria, lymphoid tissue hypertrophy, and vascular congestion and rupture were described. Regarding the ultrastructure of the detrusor smooth muscle cells, some ultrastructural changes were described following NDO induction, such as mitochondrial swelling and rough endoplasmic reticulum hypertrophy.

Several molecular factors were found to play a crucial role in the genesis of NDO and their expression depends on the NDO model and on the histological layer of the bladder wall being analyzed. It is possible to find the most relevant molecular factors explored in the included studies in Table 2 (changes in the bladder) and Table 3 (changes in neuronal tissues), along with the treatments that reportedly reverted or attenuated the NDO-related expression change. In summary, neurotrophic factors are overexpressed in the bladder of chronic SCI and PD animals and underexpressed in the bladder of MS and stroke animals. Inflammatory markers, apoptosis-related factors, and ischemia- and fibrosis-related molecules are upregulated in the bladder tissue of animals with NDO, irrespective of the NDO animal model. Purinergic, cholinergic, and adrenergic receptors are downregulated, although there are some contradictory results, as well as neuronal markers. 

### 2.4. Changes in Neuronal Tissue

Several reports indicate the occurrence of multiple changes in neuronal tissue of NDO animals, largely depending on the NDO model. As SCI was the most commonly used NDO model, the following refers to SCI, unless otherwise indicated. Following SCI, microglial and astrocyte activation was evident, along with the establishment of a pro-fibrotic scar tissue at the spinal lesion site. Gray and white matter disorganization was also reported, with neuronal cells number and their Nissl bodies being reduced. Various inflammatory cells infiltrated the lesion level. In studies using a brain injection of an active substance to induce NDO, the lesion site was reported to show gliosis and inflammatory infiltration, similar to what had been observed in SCI animals.

Many molecular factors have their expression up- or downregulated after NDO induction, depending on the NDO model and on the studied neuronal structure. It is possible to find the most relevant molecular factors explored in the included studies in Table 3, accompanied by treatments that reportedly reverted or attenuated the NDO-related expression change.

Briefly, neurotrophic factors, apoptosis-related factors and ischemia- and fibrosis-associated molecules were upregulated in the neuronal tissues of SCI animals. Inflammatory markers exhibited a tendency to increase shortly after MS induction, followed by a significant decrease from basal several weeks later. Purinergic receptors and transient channels expression showed particularly contradictory results not explained by the NDO model, location within the neuronal system, or molecular analysis technique used. Axonal growth regulators, such as MAG, Nogo-A, and RGMa, were upregulated in the lumbosacral spinal cord of the animals that suffered SCI. Expression of GFAP (a gliosis-associated protein, also used as a marker for astrocytes) was evaluated in SCI, MS, and MMC models and reports indicate it was generally upregulated, particularly near the lesion site.

## 3. Discussion

Micturition relies on intact communication between supraspinal centers, the spinal cord and peripheral neurons [1]. Connections between the pons, where the pontine micturition center (PMC) is located, and the sacral spinal cord are required for efficient voluntary control over LUT function [4]. Neurologic diseases, including SCI, neurodegenerative disorders (MS or PD), meningomyelocele, and cerebrovascular accidents, may jeopardize urinary function by causing damage to these neuronal circuits [11]. Several studies using animal models of disease have addressed and discussed changes occurring in the bladder and/or the neuronal pathways governing LUT function, contributing to a better understanding of NDO pathophysiology and potential to pinpoint possible future therapeutic targets. The present review systematically analyzed several of these studies and summarized the main findings.

### 3.1. NDO-Driven Pathology and Induction Model

Any neurological disorder that affects the micturition areas of the central or peripheral nervous system is a possible cause for NDO. We focused on NDO resulting from injury to the CNS as this was the most common situation. Our analysis shows that the vast majority of the animal models used to study NDO are based on SCI models (74%). Models reproducing neurodegenerative disorders, including Parkinson’s disease (PD) in 6% of the studies and multiple sclerosis (MS) in 10% of the articles, were less frequently reported. Animal models of meningomyelocele (6%) and stroke (4%) were reported in less than 10% of the studies scrutinized here.

#### 3.1.1. Spinal Cord Injury (SCI)

High-level SCI is followed by a period of little or no bladder reflex activity [22,24], in which the neuronal communication between LUT organs and supraspinal centers is abolished [22]. Spinal shock is gradually replaced by NDO, as a result of the neuroplastic rearrangement of micturition reflexes at the lumbosacral spinal cord [77]. These rearrangements are dependent on C-fibers [11,25,78], which undergo axonal sprouting in the bladder and lumbosacral cord [26,27,79] and lower their threshold [80], resulting in NDO [11,81]. Neuroplastic changes also likely contribute to DSD, which is frequently associated with NDO and leads to increased intravesical pressures and high volumes of residual urine, associated with a high risk of urinary infections and kidney deterioration [82]. SCI was the most-replicated pathology, possibly due to the high reproducibility and homogeneity of experimental procedures and functional outcomes. 

The spinal regions most frequently affected in human SCIs are cervical or high thoracic segments, due to abrupt flexion and/or rotation of the head or neck [83]. However, the analysed data indicate that most studies concerning urodynamic problems after SCI relied on low thoracic lesions. As any lesion occurring at the cervical region can result in respiratory compromise and is associated with a high mortality rate due to interruption of the bulbospinal respiratory drive [84,85], lesions of high thoracic or cervical segments are avoided. Instead, most studies refer to injuries between T8 and T10 that also cause NDO without affecting breathing. 

Human SCIs mostly occur due to blunt trauma (i.e., motor vehicle crash or sport injuries), where the spinal cord is damaged by an object or displaced bone and/or tissue. Thus, in SCI studies when the goal is to search for post-traumatic lesion-associated processes, repair mechanisms, or test neuroprotective treatments, the preferred method to reproduce SCI is often spinal cord contusion [85]. However, this is not the case when it comes to urological investigations. The majority of the retrieved articles in our systematic study used complete transection models [26,27,28,29,30,31,32,33,34,35,38,39,43,44,46,48,49,50,55,57,62,63] (58%), which may be explained by their ease of reproduction and the lower associated costs, as they do not require specific equipment. One could speculate that, when it comes to studying SCI-induced urinary dysfunction, the chosen method to reproduce SCI is not as important as it is in regenerative or tissue engineering studies. Nevertheless, recent studies have shown that the consequences for urinary function associated with transection and contusion models are, in fact, different [37,86,87]. Although more clinically relevant models, mild contusion models were used only in 20% of the articles in our search [37,45,59,60,61]. In these cases of mild contusion, most resorted to automatic spinal cord impactors [37,59,60,61]. These devices present reduced variability between experiments by producing a force-controlled impact, in which the amount of time that the impact tip remains on the tissue is controlled to the millisecond. Additionally, an attached force sensor precisely measures the force of the impact, which minimizes error introduced by specimen movement, and offers the possibility of immediately previewing any problem with the impact [85]. However, these systems are expensive and associated with high maintenance costs. The classical weight-drop method, used uniquely in one study [45], is more affordable and easier to use, though it does not present the same reproducibility, which may translate to a higher number of animals per study [85]. 

Other SCI protocols include incomplete sectioning of the cord with a scalpel of iridectomy scissors, the most frequent of which are spinal hemisections [42,47,52]. This model is particularly useful in studies in which the goal is to compromise a particular area of the cord. They also simulate more clinically relevant injuries when compared to complete transection, allowing comparison between injured and uninjured fibers in the same individual [88]. However, they do not consider contralateral neuroplasticity and it is more difficult to ensure reproducibility, and additional techniques are necessary to ensure injury consistency between experimental animals [85]. Compression models were the least-reported protocol to induce SCI (7%) [32,36,54,56]. These are helpful to simulate spinal canal occlusion and subsequent ischemia, which are common in clinical injuries. Based on our data, all of the compression models used an aneurism clip. This technique provides a controlled and highly reproducible injury. It is affordable and presents the possibility of controlling lesion severities by changing the force exerted in the clip and the amount of time the lesion lasts. However, compression models are less controlled than automatized contusion protocols [85]. 

#### 3.1.2. Multiple Sclerosis

MS was the second most-reported animal model of NDO (10% of the studies) [65,66,67,68,69]. MS is the leading cause of non-traumatic disability affecting the CNS, described as a neurodegenerative auto-immune disorder causing progressive neural demyelination and axonal degradation with a typical relatively early onset [5]. The consequences of MS for LUT function are thought to be attributed to spinal cord demyelination, which likely provokes imbalances between the inhibitory and excitatory neurotransmission between the spinal and supraspinal centers controlling the micturition reflex [89]. MS-related urinary impairments are variable, and likely correlated to the severity of MS phenotype, with detrusor overactivity noted in 50% to 90% of patients, whereas detrusor areflexia is observed in 20% to 30% [90]. 

Currently, the most commonly used animal model to study MS is the EAE mouse. In these animals, autoimmunity to CNS components is induced through the administration of myelin peptide fragments, which induces a rapid autoimmune reaction directed to the myelin sheath [91]. Our search identified this model as the most prevalent used in urologic investigations [66,68,69], using PLP_139–151_ [66,69] or MOG_35–55_ peptides [68] to initiate an auto-immune response. The use of each of these molecules induces distinct phenotypes with differences in regional/tract specificity, the kinetics of demyelination, and motor neuron involvement [91]. The limitation of this model is related to the discrepancies in the pathogenesis of EAE compared with human MS, as these models are poor in terms of providing information about disease progression and the role of specific T cells in MS pathogenesis. Furthermore, sex- and strain-based differences are observed in the clinical course of EAE, stressing the importance of careful choosing of experimental animals to be used in terms of age and sex.

Another model used to induce MS was the CIE mouse [65,67]. In this case, the MS phenotype was induced by the injection of mouse hepatitis virus (MHV) in a single intracranial injection. The pathology progression is contingent on the amount of the virus introduced, the age of the animal, and the strain of the murine coronavirus used, which permits the control of phenotype progression. The CIE progression phenotype is more similar to the human condition, which constitutes the golden advantage of this model. 

#### 3.1.3. Parkinson’s Disease

PD models were used in 5% of the analyzed studies [64,71,73]. PD is a neurodegenerative disorder characterized by progressive degeneration of dopamine-producing neurons in the substantia nigra of the midbrain. Together with motor symptoms, PD patients also suffer from lower urinary tract symptoms, present in 38 to 71% of the diagnosed patients [92], most frequently urgency and nocturia [93]. Loss of dopamine in the substantia nigra leads to selective depletion of the same transmitter in the striatum, accompanied by a reduction in the expression of D1 receptors in the same locations. In normal conditions, D1 receptors are involved in the inhibitory mechanisms that control storage periods [94]. Therefore, loss of D1 receptors leads to incontinence. Other PD-like pathologies, such as lesions of basal ganglia, also result in loss of voluntary control over the micturition reflex, leading to uninhibited detrusor contractions at low bladder volumes [92,94]. 

PD is a multifactorial disease. Most cases are thought to be sporadic, but specific genetic mutations have been linked to familial PD. Animal models for PD investigation can be classified into toxin or genetic models. Toxin-based models induce fast degeneration of the nigrostriatal dopaminergic neurons. In our search, the toxins used to induce PD were 1-Methyl-4-phenyl-1,2,3,6-tetrahydropyridine (MPTP) [73] or 6-hydroxydopamine (6-OHDA) [71]. Due to their structural similarity to dopamine, these toxins are absorbed by dopaminergic neurons through the dopamine transporter, causing cellular degeneration of these cells [95]. Toxin models are preferred when the goal is to study the consequences of the disease, including urinary dysfunction, rather than its onset, since they are easy to reproduce, and present reduced costs. However, toxin-based models do not fully recapitulate human PD, which has a slow and progressive onset. In these regards, genetic models are more suitable, as they provide a more realistic, human-like disease onset, offering tools to study the molecular mechanisms associated with pathology onset. Nevertheless, our search found just one study relying on genetic models, in which the GM2 gene was deleted in mice [64]. 

#### 3.1.4. Meningomyelocele

Meningomyelocele was investigated in 6% of our retrieved articles [74,75,76]. This pathology is the most severe type of spina bifida, a congenital neurological abnormality occurring when the spinal cord does not form properly due to defective closure of the caudal neuropore of the neuronal tube. As the spinal nerves controlling bladder function do not form correctly, meningomyelocele is accompanied by neurologic bladder symptoms [96], including NDO and DSD. To induce meningomyelocele, studies resorted to pregnant female rats, intragastrically injected with retinoic acid on embryonic day 10. This model is capable of reproducing the entire spectrum of severity observed in human meningomyelocele, ranging from exposure of the cord with intact neural elements to complete cord destruction [97].

#### 3.1.5. Cerebral Vascular Accidents

Cerebral vascular accidents were the least-used animal NDO models in our search [70,72]. However, more than half of stroke patients, either ischemic or hemorrhagic, report symptoms of urinary dysfunction, including urinary urgency, frequency, and urge incontinence. The presence of DSD is also encountered [98]. These urodynamic symptoms may be present within 72 h of the cerebrovascular accident and, in 30% of the patients, within four weeks after that time point [99]. In our search, we found two methods to induce stroke: middle cerebral artery occlusion (MCAO) and enzymatic induction of cerebral hemorrhage. The MCAO model, used to study ischemic stroke [70], is achieved by the insertion of a filament in the middle cerebral artery, which is removed afterwards. This produces a transient ischemia followed by the restoration of blood circulation, as it happens in humans. This method avoids the need for craniotomy and its possible negative effects on blood–brain barrier permeability and intracranial pressure [100]. However, MCAO may cause subarachnoid hemorrhage, tracheal edema, and paralysis of the muscles of mastication and swallowing if damages in the external carotid artery occur [100]. The other method referred to is the induction of cerebral hemorrhage. In this case, the hemorrhage is induced by a collagenase injection in the hippocampal CA1 region [72]. Collagenase enzymatically disrupts the basal lamina of blood vessels, causing an active bleed into the surrounding tissues that generally evolves over several hours. Both methods can be adapted to injuries in any brain region. 

### 3.2. Animal Species

Our search demonstrated that rodents were used in 95% of the retrieved articles concerning NDO. Rats were the most commonly used (73%), followed by mice (22%). Rats have the advantage of low maintenance costs, ease of care, and a well-studied anatomy [101]. Their bigger size, when compared to smaller rodents, allows for more complex surgical interventions, which is particularly important in models based on the physical lesioning of CNS areas. Several established behavioral tests, which are used to assess the loss and recovery of neurologic deficits, are better adjusted for rats than other rodents. Concerning urodynamic testing, the majority of established techniques are better studied and established in rats, providing superior testing outcomes and numerous sources of comparison [102]. However, mice models are becoming more popular and increasingly implemented in NDO studies. Morphologically, the mouse bladder appears to be more similar to humans, but the urodynamic properties of the mouse LUT have not been characterized as well as those of rats [103]. Mice offer the possibility of generating genetically modified models and have higher reproductive rates and low maintenance costs. The disadvantages of using mice are related to their smaller size, which poses problems for several induction protocols and urodynamic recordings and urethral electromyography [103]. 

Despite the benefits of using experimental animals to investigate NDO pathophysiology and test new therapeutic approaches, the results should be approached with caution. It is important to note some morphological and physiological differences. In rodents, the prostate is not encapsulated within a well-formed prostatic fascia [104]. Additionally, the architecture of the pelvis and pelvic floor corresponds directly to the quadruped locomotion of rodents, which is different between the two species. Functionally, there is evidence that detrusor contraction in rodents is dependent on ATP acting as a neurotransmitter, whereas in humans, it is mediated by acetylcholine [103]. These differences may affect the functional outcomes [104]. 

Other animal models, such as rabbits and non-human primates, may provide a more physiologically relevant evaluation of outcomes compared to rodents, particularly considering the similar size of the spinal cord, comparable neurological damage mechanisms, and higher anatomical parallel. However, the use of these non-rodent animal models is limited by maintenance costs and strict ethical requirements. Accordingly, our systematic search encountered three studies using rabbits [33,41], and one using the marmoset [73]. No studies with primates were reported.

### 3.3. Animal Sex

Our analysis demonstrated that more than 70% of the studies favored females to induce NDO. This is likely related to the feasibility of transurethral catheterization and manual bladder emptying, since the male urethra is surrounded by the prostatic gland, which makes abdominal compression and bladder manipulation more difficult in males [33,104]. Nevertheless, sexual dysmorphism in micturition behavior should be accounted for. In addition, one should not forget that some human pathologies might be more prevalent in one **sex** than the other, making data obtained in studies using only female or male animals more difficult to clinically translate. Experiments concerning the effect of the estrous cycle on rat bladder contractility have pointed to a more responsive behavior of females bladders [105] compared to males in response to cholinergic stimulation. This likely reflects **sex**-related differences in bladder expression of different subtypes of muscarinic and adrenergic receptors [106,107,108]. Accordingly, the cholinergic neurotransmission is predominant in the male bladder, while the purinergic component is prevalent in females [109]. Sex differences were also noted in expression of acid-sensitive ion channels (ASICs) and transient receptor potential vanilloid type 1 (TRPV1), both key channels for normal and pathologic bladder function [110]. These molecular discrepancies are likely reflected in bladder function. 

Micturition patterns are also different between male and female experimental animals. Male voiding consists of a fast spike-like urine flow, whereas female voiding is ongoing but interrupted for short periods when bladder pressure is increased. The maximum flow rate is lower and voiding period is shorter in female rats as compared to male rats [111]. These dimorphic micturition patterns in rats might be attributed to the different nature of the perineal muscles of the EUS, less prominent in females [112]. Though these differences are considered to be of minor relevance in normal function, they might have a significant impact in pathologic conditions. To understand the real impact of sex on pathologic LUT function, research studies would benefit from using both male and female animals. However, this was only the case in two studies [64,73], both concerning PD models. 

### 3.4. Urodynamic Recording

Changes in LUT function are evident after induction of SCI, MS, PD, meningomyelocele, or stroke. Animals present typical NDO symptoms, including increased voiding frequency, basal pressure, maximum voiding pressure, threshold pressure, and high amounts of residual urine. As result, the voided volume per contraction is reduced and a significant decrease in voiding efficiency is evident. Some studies also reported the presence of DSD. The majority of the retrieved articles evaluated the consequences on LUT function by using urodynamic recording techniques. 

For decades, the gold standard method to perform urodynamic evaluation in animals has been using cystometry under urethane anesthesia. This drug is the most-used anesthetic for urodynamic recording, as it is recognized as the most preservative of micturition reflexes. Nevertheless, urethane interferes with urethral sphincter activity, resulting in reduction in voiding efficiency and increase in post-void residual volume [113,114,115,116,117]. Moreover, urethane anesthesia is limited to terminal procedures, due to its adverse post-operative health effects and carcinogenic risks [118]. One could speculate that recent papers would resort to better anesthetic options, but urethane remains the main choice for urodynamic evaluations [26,27,28,37,41,43,51,57], as it has low associated costs, it is easy to deliver, and there are abundant published references using this anesthetic for cystometries [102,119].

Fewer studies have used zolotyl anesthesia as an alternative for urethane [53,58,61,74]. Zolotyl is a combination of tiletamine and zolazepam and was used in 8% of the articles in our search. Zolotyl produces a smooth conscious sedation, characterized by a rapid induction period, together with excellent muscle relaxation with a wide safety margin, and smooth recovery [120]. One article included in our search used chloral hydrate as an alternative to urethane to perform cystometries [54]. This anesthetic is no longer recommendable to use, due its toxic components. 

In an attempt to overcome the negative effects of anesthetics on LUT function, awake cystometries have arisen as a popular method to record bladder and urethral function in rats and mice. Unlike anesthesia protocols, in which environmental cues and diurnal variations are suppressed, it is necessary to consider that cystometries in awake conditions are influenced by external factors, such as light and noises. Furthermore, as rodents are nocturnal, the experiments must be performed during night time, or animals must be acclimatized to inverted cycles of light [102]. The variety of awake recording systems range from restrained to freely moving animal approaches [121]. In restrained animals, it is easier to manage the position of the intravesical catheter, and to prevent the occurrence of urodynamic artifacts. However, despite pre-testing habituation to the cystometry stations, restraining may cause high levels of stress, which can increase sympathetic activity, favoring storage and potentially prolonging the time of bladder filling until micturition [122]. Unrestrained conditions, using metabolic cages, closely resemble physiological conditions and are assumed to better record LUT function [123], but studies using this approach are still scarce.

For urodynamic assessment in experimental animals, it is necessary to place an intravesical catheter that allows for saline injection and/or recording of bladder contractions. These catheters can be placed acutely or be indwelling. Acutely placed catheters are suitable for terminal procedures when the recordings occur right after implantation surgery and the animal is euthanized immediately after recording. There are, however, disadvantages linked to the use of acute catheters, such as postoperative pain and the fact that the anesthetics used in the surgical procedure might affect LUT activity [102,122]. An alternative for this is the use of chronic indwelling catheters and electrodes for urethral electromyography, which can be externalized on the animal’s dorsum and maintained for large periods, permitting animal stabilization after surgery. Additionally, they also allow the testing of the same animal several times during the experimental protocol, eliminating inter-animal variability and reducing the number of animals required [124]. However, these systems are associated with a high maintenance cost and complex post-operative care to maintain the functionality of the externalized components for extended periods and the wellbeing of the animals [102,124]. 

While urodynamic recording is the only method that can objectively assess lower urinary tract function, non-invasive methods, including the voiding spot assay, were also reported in our search [64,65,67,68]. The voiding spot test has the advantage of being minimally invasive, inexpensive, and easy to implement. However, the urodynamic data are poor, only recording voided volumes and spatial and temporal organization of urinary spots [102]. Surprisingly, this was the preferred method to evaluate bladder changes after induction of neurodegenerative disorders [64,65,67,68].

Surprisingly, a significant portion of retrieved studies did not present any urodynamic data [29,30,36,44,45,49,50,60,62,66,69,73,74,75,76]. This was quite unexpected for studies with a focus on urinary dysfunction and NDO. One could speculate that these studies used animal models that are already established, so their effects were already known and described. These studies focused on other aspects of the disease than urinary function, including molecular alterations. In fact, the lack of any evaluation of LUT function was seen in studies using animal models of myelomeningocele, in which urodynamic recording would be difficult to perform. 

### 3.5. Changes in Bladder and Neural Tissue Morphology

Selected studies highlighted significant findings regarding bladder and neuronal tissue morphology. Bladder tissue was generally more fibrotic in NDO animals, in tandem with findings in humans [125]. Histologically, bladder fibrosis is described as an increase in connective tissue elements, particularly in the detrusor, where collagen fibers heavily surround smooth muscle cells. These changes are driven by several molecular factors, which are upregulated in NDO bladders, and represent tissue remodeling following DSD and consequent bladder volume load increase. This functional obstruction also leads to detrusor smooth muscle hypertrophy, chronic inflammation, and edema. All these features result in an increase in bladder weight, reflecting bladder wall thickening [59]. Smooth muscle ultrastructural changes after SCI-inducted NDO were also found in the retrieved studies, such as mitochondrial swelling and endoplasmic reticulum hypertrophy [54], consistent with smooth muscle hypertrophy and increased intensity of bladder contractions. The mucosa, particularly the urothelium, also undergo plastic changes, shown to contribute to impaired urinary function in NDO models [126].

Concerning neuronal tissue, various CNS and PNS structures are affected, depending on the NDO model. SCI was the model that presented greater morphological changes, with formation of fibrotic scars at the injury site, associated with recruitment microglia, astrocytes, macrophages, and other inflammatory cells. These cells eventually fill the injury core and are involved in complex crosstalks to repair the injured tissue but prevent axonal regrowth [127]. Because SCI is the most-used method to induce NDO, the considerations below mostly refer to SCI.

### 3.6. Molecular Factors

Molecular changes in the bladder and neuronal tissue after NDO induction are, respectively, presented in Table 2 and Table 3. These variations were detected through either protein or RNA analysis. The majority of data gathered was obtained from SCI studies.

#### 3.6.1. Neurotrophic Factors

Neurotrophic factors are growth factors that play a critical role in neuron survival and regeneration, including nerve growth factor (NGF), brain-derived neurotrophic factor (BDNF), and glial cell-derived neurotrophic factor (GDNF) [128,129]. NGF is a small molecular weight protein, involved in urinary dysfunction in several contexts, including SCI [130,131]. In the bladder, NGF is secreted by smooth muscle and urothelial cells [132,133,134], and its levels are increased in response to inflammation or denervation [135]. In animal models of NDO, bladder NGF levels also vary, increasing after SCI [29,46,48,56] and being reduced in CI and MS animals [66,70]. In the latter, the time point studied referred to chronic stages of disease progression and it is not possible to exclude increased NGF levels in the acute phase of the MS model [66]. Importantly, while it is possible to observe that, as in cystitis [134,136,137], SCI-induced NDO courses with high NGF levels [138,139,140], the same does not happen in MS models. While this likely reflects different pathophysiological mechanisms for NDO, the precise reasons can only be speculated at present. Importantly, high levels of bladder NGF coursed with upregulation of the phosphorylated form of the high-affinity NGF receptor TrkA, which was also observed in PD models [29,64].

In neuronal structures, NGF was also upregulated after NDO induction, when quantified in nervous system structures, such dorsal root ganglia, supraspinal neuronal voiding centers and the spinal cord [48,53,57,61,72]. Nevertheless, it is important to point out that this analysis was not performed on an SCI or on an MS model. Regarding BDNF, protein expression was increased in SCI animals, both in the bladder and the spinal cord [27,61], but variations of its levels in other models were not found. Changes in GDNF levels were only reported in an MS model, in which the bladder contents were found to be reduced [66]. Such changes in neurotrophic factors are likely involved in the abnormal axonal sprouting resulting in expansion of C-fibers in the bladder wall and lumbosacral spinal cord, a key event in NDO development and maintenance [12,26,27,80].

#### 3.6.2. Inflammatory Mediators

Changes in inflammatory molecules, including pro- and anti-inflammatory cytokines, were described in articles using MS animal models. These mediators, such as IL-2 and TGF-β, were dramatically increased in the bladder tissue [59]. In neuronal tissue, the variation in expression levels of cytokines is complex to analyze. Inflammatory cytokines were increased after 1 week but decreased 10 weeks after MS induction [65,67]. The first week period in animal MS models may represent an acute immune event, linked to inflammatory demyelination. The reduction at 10 weeks likely reflects modulation of immune responses. These changes coursed in NDO installation and likely reflect changes in immunological activation associated with MS.

#### 3.6.3. Apoptosis-Related Factors

Apoptosis is the process of programmed cell death, involving several players in complex pathways, including enzymes such as caspases. In animal models of SCI, Caspase-3 was found to be activated at the injury site, since trauma and ensuing events lead to cell death of resident and invading cells [127]. After traumatic SCI, spinal tissue at the injury site undergoes major transformations. Healing is a complex process that results in tissue remodeling, which seals the injured location. Traumatic SCI causes direct tissue destruction (compression, laceration, shearing of the cord) that results in profound histological modifications at the injured location [141,142,143]. This is followed by production of free radicals, lipid peroxidation, altered ATP production, invasion of peripheral immune cells (including neutrophils, lymphocytes, and monocytes) due to breakdown of the blood–brain barrier, activation of resident astro- and microglia, and neuronal and glial apoptosis, all of which contribute to further damage of the injured area [142]. The final step consists of the formation of a glial scar, highly repulsive to axonal growth, preventing appropriate rewiring, reestablishment of connections between supraspinal centers and lumbosacral neurons and, ultimately, full recovery [142]. While apoptosis is central at the injury site within the spinal cord, we found no reference to a direct link with NDO development or maintenance. No studies addressed the presence of pro-apoptotic elements in the bladder of NDO animals.

#### 3.6.4. Muscarinic Receptors

Muscarinic receptors play an important role in detrusor contraction and they can be found in the detrusor layer and the mucosa, participating in the urothelium–detrusor crosstalk and regulating detrusor contraction [144,145]. The mRNA levels of M2 and M3 muscarinic receptors in the bladder mucosa of SCI animals (6 weeks after lesion) were downregulated, but only M2 protein levels were reduced when compared to controls [44]. Another study with rodents, not included in this review [144], showed an increase in M2 subtype transcript in the bladder mucosa 2 weeks after SCI, returning to basal by 4 weeks. There was no similar pattern in the M3 subtype. Detrusor protein expression of M2 receptors increased during chronic SCI period and the M3 subtype was downregulated [56]. In animals with cerebral ischemia, M3 was downregulated [70], but there are conflicting observations regarding M2 levels, possibly reflecting different analytic techniques [70]. Changes in the expression of muscarinic receptors may underly the lack of response of patients to anti-muscarinic therapy. This is relevant as treatment of NDO is typically initiated with anti-muscarinic drugs [9,16]. If patients do not respond to low amounts of these drugs, the dosage is increased, but only refractory patients will receive botulinum toxin A as the last-resort treatment [146,147].

#### 3.6.5. Adrenergic Receptors

In terms of adrenergic receptors, the retrieved studies documented changes in their expression in the bladder of SCI animals presenting NDO. In the clinical setting, α1a adrenergic receptor (AR) antagonists have been used in multiple pathologies, such as prostatic benign hyperplasia [148] and DSD after SCI, to produce muscle relaxation and decrease urethral sphincter pressure and obstructive symptoms [60]. However, this therapy is not always fully effective, which likely reflects the downregulation of α1a adrenergic receptor expression after SCI [60]. The expression of β2-adrenergic receptors in the bladder was also studied in the context of SCI and a reduction in the bladder levels of β2-adrenergic receptors was found [56]. 

#### 3.6.6. Purinergic Receptors and Transient Receptor Potential Channels

The importance of P2X and TRP receptors in urinary function is well established [149]. We found two contradictory perspectives on the expression of P2X purinergic receptors and TRP channel expression in SCI animals. Concerning the expression of these receptors in DRG cells, four studies [35,46,49,63] reported upregulation of TRP and P2X receptors, while another study [45] reported TRP/P2X pathway elements’ downregulation. Similarly, in the bladder, three studies [30,51,56] showed upregulation of TRP and P2X elements, while one reported downregulation [45]. These different observations likely originate from different methodologic approaches, as the majority of available studies refer to the upregulation of these ion channels as key events to explain enhanced excitability of C-afferents, known to be a driver of NDO development and maintenance in SCI [11,12,78]. 

#### 3.6.7. Neuronal Markers

Analysis of β-III-tubulin, a pan-neuronal marker, in 9-week SCI rats shows upregulation of this protein in bladder tissue, indicating the occurrence of hyperinnervation in the bladder wall and demonstrating neural plasticity and compensatory axonal regeneration [36]. This agrees with increased bladder levels of neurotrophins, such as NGF, which induce axonal growth and branching [130,131]. 

In the nervous system of NDO rats, the expression of several neuronal markers (namely CRF, GAD2, NF200, TH, VAChT, and VGLUT) was found to be generally downregulated, due to denervation associated with SCI, MMC, and PD, the latter more evident in the substancia nigra and ventral tegmental area [40,47,49,64,73,74]. In contrast, the expression of CGRP, a marker of sensory innervation, was upregulated in the lumbosacral spinal cord and L1 and L6 DRGs in SCI animals, in tandem with what has been described in the bladder [26] and coursing with levels of spinal NGF [140]. 

#### 3.6.8. Ischemia- and Fibrosis-Related Molecules

This category includes not only molecular factors that play a critical role in the setting of fibrosis, such as CTGF, FGF, and TGF, but also a broader group of molecules responsible for ischemic response, such as HIF and VEGF. All were upregulated in the bladder and neuronal tissue in SCI and MS models. This indicates that, in both conditions, NDO development and maintenance are associated with intensive tissue remodeling [59]. 

Astrocyte-derived chondroitin sulphate proteoglycans (CSPGs)—phosphacan and neurocan—were also included in this group, since they are the central extracellular matrix components of the spinal fibrotic scar that seals the injury site after SCI [28,127]. The levels of CSPGs are highly increased at the injury site after SCI [142,150], correlating with upregulation at the same location of S100, a glial marker [41], consistent with the accumulation of glial cells and scar formation [142,150]. Importantly, CSPGs are also elevated in in segments distant from the scar [28,151], indicating a widespread response to SCI. While this upregulation of CSPG content is exhuberant at the injury site, it is more controlled and restricted in segments distant from the injured tissue, as only the expression of specific CSPGs is changed in a time-dependent manner [28,151]. CSPGs are known to be involved in axon guidance regulation [152] and it is possible that this lumbosacral upregulation might be linked to the establishment of new neuronal circuits responsible for abnormal bladder function after SCI. 

#### 3.6.9. Myelin-Associated Proteins

This group of molecules can be divided in two different clusters: proteins associated with the myelin sheath, such as myelin basic protein (MBP), and myelin-associated inhibitory proteins (MAIs)—MAG, Nogo-A, and OMgp. MBP was downregulated in the spinal cord, both after SCI and MS. MBP downregulation possibly reflects loss of myelin sheaths due to apoptosis of neurons and oligodendrocytes secondary to SCI [62], while its downregulation in MS models can be explained by CNS demyelination. Concerning MAI expression in the lumbosacral cord, levels of Nogo-A were transiently upregulated, without changes in MAG and OMgp, after thoracic SCI [28]. Changes in the expression of these guidance molecules may well be linked to neuroplastic events leading to NDO establishment. 

## 4. Conclusions

NDO is a common consequence of neurologic injuries, with a tremendous impact on the quality of life of affected patients. Animal models of NDO have been critical for understanding the pathophysiology of the disease, as well as to study potential for recovery and implement new therapeutic targets for affected patients. In this review, we describe the currently used animal models to study NDO, and discuss them in terms of species, sex, urodynamic recordings, and molecular alterations observed in bladder and neuronal tissue. Despite the heterogeneity of NDO onset, the vast majority of studies concerning molecular mechanisms associated with this pathology are based on traumatic SCI models. However, NDO is also a consequence of several progressive diseases such as neurodegenerative disorders, meningomyelocele, and stroke, about which there is much less information. It is important that future studies focus on these disorders to provide a better understanding of the pathophysiological mechanisms leading to NDO, which is important for the development of new therapies targeting these patients’ quality of life. The list of molecular changes found in the present review is vast and includes the upregulation of inflammatory mediators, molecular markers of fibrosis. Moreover, there is also significant evidence of neuronal plasticity with increased expression of neuronal receptors, neurotrophins, and myelin-associated proteins both in the bladder and neuronal tissue, supporting the wide range of neuroplastic events that result in NDO. While it is difficult to grasp and integrate the enormous number of published results, it is clear that NDO pathophysiology is complex and, consequently, its treatment and management is difficult. Like other researchers [18] and following the present review, we propose that many key players are active and interacting at different stages of disease progression. It is likely that future interventions will result from the combination of different drugs simultaneously targeting different molecules. Future research should use comprehensive strategies, possibly automated, to identify synergistic changes and key events that could be therapeutically targeted. 

## 5. Materials and Methods

### 5.1. Literature Search

The present systematic review was elaborated following the PRISMA 2020 checklist [153]. We aimed to analyze scientific articles that addressed molecular changes associated with neurogenic detrusor overactivity. On 19 September 2022, the search was conducted in three databases: PubMed Central (via PubMed), and Medline and Embase (via Scopus). The following query was used: ((Animal model) OR (rat) OR (mice) OR (rabbit) OR (pig)) AND ((Neurogenic detrusor overactivity) OR (neurogenic bladder)). No filters were used, and the search was limited to articles published between 1 January 2012 and 19 September 2022. The year 2012 was chosen as a reference as it was the year in which some automated devices for spinal contusion became commercially available [154]. This search generated 648 results.

### 5.2. Selection

The studies retrieved were imported to Endnote and duplicated articles were excluded. The remaining articles were then imported to the Rayyan platform, and the remaining duplicates (not detected by Endnote) were identified and excluded. The resultant articles (365) were submitted to title and abstract screening, independently conducted by two investigators. The inclusion criteria were: (1) studies including an animal model of neurogenic detrusor overactivity; (2) studies including at least one molecular analysis technique; and (3) studies published in English. The exclusion criteria were: (1) non-original studies, such as reviews, conference abstracts, and editorials; (2) studies conducted in humans, such as case reports and clinical trials; (3) in vitro studies; (4) absence of data on molecular alterations; (5) studies using an SCI model which merely present molecular results obtained from animals euthanized 14 days after SCI induction (acute SCI); (6) studies focusing on NDO of peripheral origin; and (7) impossibility of obtaining the full-text article (even after contacting the authors). All articles were submitted to full-text screening if no inclusion criteria were absent and if no exclusion criteria were met.

To assess the risk of bias in our work, SYRCLE’S risk of bias tool was used. This checklist was adapted from the Cochrane risk of bias tool and adjusted for experimental animal studies. The tool was developed by Hooijmans et al., and focuses on evaluating selection bias, performance bias, detection bias, attrition bias, and reporting bias in animal experimental studies [155]. The Cochrane risk of bias (Rob) checklist was also consulted [156]. 

### 5.3. Data Extraction

Outcomes for which data were sought were the following: (1) study characteristics (author and year of publication); (2) used model of neurogenic detrusor overactivity; (3) used animal species; (4) animal sex; (5) model induction method; (6) urodynamic findings; (7) changes in bladder tissue; (8) changes in neuronal tissue; and (9) therapies and mechanisms identified. If any of these study characteristics were not evident from full-text analysis, authors were contacted. Basic study characteristics, such as the animal sex, are described as unknown if there was no answer from the authors. All gathered data have been included in Table 1. Two independently working reviewers extracted the most relevant data from every included article. No automatic tools were used. 

## Figures and Tables

**Table 1:** Summary of included studies in this systematic review. The data were extracted and sorted into the following categories: model, species, sex, induction method, urodynamic findings, changes in bladder tissue, changes in neuronal tissue, and therapies/mechanisms identified. NDO: neurogenic detrusor overactivity; SCI: spinal cord injury; MAG: myelin-associated glycoprotein; OMpg: outer membrane protein G; RGMa: repulsive guidance molecule A; NGF: nerve growth factor; DRG: dorsal root ganglion; Trka: tropomyosin receptor kinase A; AKT: protein kinase B; TRPM4: transient receptor potential cation channel subfamily member 4; NF200: neurofilament 200; S100: S-100 protein marker; TRPV1: transient receptor potential cation channel subfamily V member 1; M3: muscarinic receptor 3; TGFB1: transforming growth factor beta 1; BoNT-A: botulinum toxin-A; DSD: detrusor sphincter dyssynergia; BDNF: brain-derived neurotrophic factor; ASIC: acid-sensing ion channel; RTX: resiniferatoxin; GAP43: growth-associated protein 43; CGRP: calcitonin gene-related peptide; IPHFO: duration of intra-luminal pressure at high-frequency oscillations; GFAP: glial fibrillary acidic protein; PGE2: prostaglandin E2; HIF: hypoxia-inducible factor; TGF: transforming growth factor; bFGF: basic fibroblast growth factor; NVC: non-voiding contraction; CRF: corticotropin-releasing factor; EUS: external urethral sphincter; PGP. 9.5: protein gene product 9.5; TH: tyrosine hydroxylase; JNK: c-Jun N-terminal kinase; SNAP-25: synaptosomal-associated protein, 25kDa; ATF3: activating transcription factor 3; TRPA1: transient receptor potential cation channel subfamily A member 1; P2X: purinergic receptors; PTNS: percutaneous tibial nerve stimulation; 5-HT: 5-hydroxytryptamine; VGLUT2: vesicular glutamate transporter 2; GABA: gamma-aminobutyric acid; GAD2: glutamate decarboxylase 2; KV1.4: potassium channel Kv1.4; CD11: integrin beta chain-2; SMA: smooth muscle actin; ki67: antigen KI-67; TUNNEL: terminal deoxynucleotidyl transferase dUTP nick end labeling; ICC: interstitial cells of Cajal; PAG: periaqueductal gray; PMC: pontine micturition center; GAPDH: Glyceraldehyde-3-Phosphate dehydrogenase; TGN: tissue gene nerve; VEGF: vascular endothelial growth factor; iNOS: nitric oxide synthase; VTA: ventral tegmental area; IFN: interferon; MBP: myelin basic protein; CTGF: connective tissue growth factor; TGF: transforming growth factor; EAE: experimental autoimmune encephalomyelitis; CNS: central nervous system; IL-B: interleukin beta; CIE: coronavirus-induced encephalitis; MS: multiple sclerosis; Panx1: pannexin 1; cx34: gap junction alpha-1 Protein; SCF: Skp1-cullin 1-F-box; EP2/3: prostaglandin receptors; MCAO: experimental middle cerebral artery occlusion; PD: Parkinson´s disease; 6-OHDA: 6-hydroxydopamine; MPTP: 1-metil-4-fenil-1,2,3,6-tetraidropiridine; VaCHT: vesicular acetylcholine transporter; MMC: meningomyelocele; NeuN: neuronal nuclei.

Study	Model	Species	Sex	Induction Method	Urodynamic Findings	Changes in Bladder Tissue	Changes in Neuronal Tissue	Therapies/Mechanisms Identified
Chambel et al., 2022 [28]	SCI	Rat	Female	Complete cord transection T8/T9	Cystometry under urethane anesthesia: at 7 dpi, bladder contractions were abolished, while at 28 dpi, the NDO was established, with the parameters of frequency, amplitude, basal pressure, and peak pressure augmenting in comparison with intact rats.		Changes in lumbosacral expression of axonal growth regulators: neurocan, phosfacan, nogoA, MAG, OMgp, RGMa: Increase at 7 dpi and decrease to basal levels at 28 dpi -Decrease in the DRG of those specific receptors: CSPG- and MAI-receptors.	NGF regulates the expression of receptors for axonal guidance cues in DRG neurons. When exposed to high levels of NGF, some repulsive cues decrease its expression.
Zhang et al., 2019 [29]	SCI	Rat	Female	Complete cord transection T8/T9 at suprasacral levels		After acupuncture treatment: -Decrease in bladder weight; -Decrease in fibrosis levels and smooth muscle cell damage; -Increase in NGF and TRka and p-akt with treatment: nerve regeneration.		Sacral electroacupuncture therapy can improve the expression of both NGF/TrkA signaling and AKT signaling in the local nerve of the damaged spinal cord and is capable of improving micturition function after injury.
Kullmann et al., 2018 [30]	SCI	Mouse	Female	Complete cord transection at T8–T9		-TRPM4 levels largely increase after SCT—earlier time points 3–7 days; -Blocking of TRPM4 activity (by an antagonist) reduces spontaneous muscle activity.		Pharmacological blocking of TRPV4 (9-phenanthrol-ANTAGONIST) reduces spontaneous phasic activity developed after SCI, showing its involvement in SCI-induced bladder neurogenic activity.
Gotoh et al., 2020 [31]	SCI	Mouse	Female	Complete cord transection T8–T9	Awake cystometry: decreased number of non-voiding contractions in vibregon treated rats; the time required to induce the first voiding was higher.	Increase in mRNA levels of collagen, TGF-B1, FGF-2, HIF-a, VEGF-a (ischemic molecules); Vibregon treatment decreased the levels of collagen, TGF and HIF; collagen protein levels were not altered.		Vibregron (β3-adrenoceptor agonist) treatment reduces non-voiding contractions and decreases the expression levels of fibrosis- and ischemia-related molecules.
Chung et al., 2015 [32]	SCI	Rat	Male	Complete transection, or cord compression	Awake cystometry: injured animals presented detrusor overactivity, showing frequent non-voiding contractions; treatment with inosine reduced the frequency and amplitude of detrusor contractions.	-Decreased expression of synaptophysin (SYP); -Decreased expression of NF200-positive axons in bladder; -Increased TRPV1 expression. Inosine treatment attenuated these alterations.		Inosine treatment (immediate or delayed) reduces urinary complications after SCI. The mechanisms are unclear, but it is known that it is not related to muscle contractility, and maybe is through TRPV1.
Shang et al., 2019 [33]	SCI	Rat	Female	Complete transection at T9–T10	Awake cystometry: increased basal pressure,maximum voiding pressure, threshold pressure, volume of residual urine, and number of voiding contractions; decreased inter-contraction interval. All features attenuated by treatment.	Decreases expression of M3 receptor mRNA in SCI animals, and treatment decreased this expression; M3 receptor protein expression does not correlate with changes in the level of M3 receptor mRNA.		Downregulation of M3 receptor expression in the bladder wall by lentivirus-mediated RNAi can significantly decrease urinary dysfunction, targeting NDO symptoms associated with SCI.
Jia et al., 2021 [34]	SCI	Rat	Female	Complete transection at T10	Awake cystometry: increase in non-voiding contractions; botox attenuated this, more in earlier treatments than late.	-Increase in bladder weight, levels of connective tissue/fibrosis; -TGF-β1 (TGF-β1 is a major driver of human fibrotic protein) expression increased.Treatment with BoNTA decreased significantly in the early group compared with the late group; mRNA levels were in line with this.		Early BoNTA injection had a tendency to prevent bladder fibrosis and improve NDO, downregulating TGF-β1 expression.
Saito et al., 2021 [35]	SCI	Mouse	Female	Complete transection at T8/9	Awake cystometry: -Non-voiding contractions were observed at 2 weeks post injury; bladder capacity was increased at 6 weeks; -DSD was observed 2 weeks post injury, but periodic EMG reductions that produce voiding were not observed at this time point, until 4 weeks.	Increase in BDNF at all injury timepoints: higher at 2 weeks and decreases at 4 and 6 weeks, but never returning to basal levels.	Increase in mRNA levels of TRPV1, ASIC1, ASIC3-, and piezo2 DRG L6-S1.	The development of DSD might be related to changes in the expression of mechanosensitive channels such as ASICs and Piezo2; changes in these channels are accompanied by changes in BDNF expression.
Oliveira et al., 2019 [26]	SCI	Rat	Female	Complete transection at T8/9	Cystometry under urethane anesthesia: increased frequency of bladder contractions and higher peak pressures; RTX treatment attenuated this.	-Increase in bladder wall thickness; -Increase in bladder TRPV1; RTX treatment attenuated this.	-Severe loss of gray matter arrangement in dorsal and ventral horns, with white-matter disruption and several cavities; -Increase in lumbosacral spinal GAP43 and CGRP expression.	The neurotoxin RTX is capable of reducing urinary symptoms related to SCI; these effects were only seen in the bladder, not affecting the spinal cord.
Ozsoy et al., 2012 [36]	SCI	Rat	Female	Spinal compression at T8	One week after SCI, all groups presented bladder areflexia; in the severe contusion group, urinary function did not improve. Mild contusion rats presented better scores following the third week after lesion.	Detrusor hyperinnervation: increases in β-III-tubulin.		Bladder function was significantly worse following severe compared to moderate compression injury.
Munoz et al., 2017 [37]	SCI	Rat	Female	Spinal contusion at T8/T9: mild and severe	Cystometry under urethane anesthesia: after mild contusion, mice presented increased inter-contractile intervals, high number of non-voiding contractions, and longer duration of the IPHFO (duration of intra-luminal pressure high frequency oscillations). Severe contusion mice showed a high NVC frequency, practically absent IPHFO events, and small voiding volumes.		-GFAP increase (astrogliosis) and microglial activation in both groups 4 weeks after SCI (at injury level).	Independent of the impact intensity, a contusion SCI increases microglia and astrocyte infiltration into the injured region without a strong correlation with locomotor impairment; bladder dysfunction is worse in severe than mild contusion.
Wada et al., 2018 [38]	SCI	Rat	Female	Complete transection at T9–T10	Awake cystometry: the time to the first non-voiding contraction was significantly prolonged after both 0.1 and 1.0 mg/kg of intravenous administration of SC51089; other parameters were not altered.		Increase in PGE2 concentration of the L6 and S1 spinal segments; the mRNA expressions of EP1 receptors of the L6 and S1 spinal cord and DRG from SCI rats were increased.	SC51089-prostaglandin 1 receptor antagonist PGE2-induced EP1 receptor activation is involved in the initiation of C-fiber excitation to trigger NVC in SCI rats.
Wada et al., 2017 [39]	SCI	Rat	Female	Complete transection T8–T9	Awake cystometry: inter-contraction interval, bladder capacity, and bladder compliance were significantly increased in SCI animals treated with combination therapy, and not monotherapies; the time required for the first NVC was significantly prolonged in the oxybutynin and combination group.	Type 3 collagen, HIF-1a, TGF-β1, and FGF-β (actors involved in tissue remodeling and hipoxia) were reduced in oxybutynin and combination therapy; in mirabegron therapy, the expression of mRNA of HIF-1α and TGF-β1 was significantly reduced compared to controls.		The combination therapy of an anticholinergic agent (oxybutynin) and a b3-adrenoceptor agonist (mirabegron) elevated the bladder elastin level, reduced NVCs, and increased bladder compliance to a greater extent than the monotherapy of either drug in SCI.
Shneider et al., 2019 [40]	SCI	Rat	Female	Incomplete or complete transection at T8	Awake cystometry: bladder was areflexic the first week after injury; over the following 3 weeks, maximum detrusor pressure constantly increased, exceeding the baseline measurements at 4 weeks; reduction in voiding rates and urine volumes; reduction in bladder compliance; development of DSD. Treatment with aniti-Nogo-A improved several urodynamic parameters.		After 4 weeks, animals treated with vehicles showed decrease in CRF-positive innervation of Lam X; animals treated with anti-Nogo-A antibody presented values similar to intact rats; in the IML region, both injury groups showed a reduced CRF-positive fiber density; anti-Nogo-A antibody-treated rats showed a trend for higher GABAergic values, GAD2 mRNA-positive cells decreased in L6-S1.	Anti-Nogo-A antibody treatment improved urodynamic and electrophysiological parameters in SCI animals, namely a pronounced recovery of the physiological EUS function during voiding. This is likely due to protection of spared descending fibers from the PMC sprouted below the level of the injury in a specific target region, Lam X, thereby restoring functional input from the key bladder control system.
Sadeghmousavi 2022 [41]	SCI	Rabbit	Male	Spinal cord cauterization at T12-L1	Cystometry under urethane anesthesia: increase in bladder capacity, decrease in leak point pressure, increase in leak point volume.	Lymphoid tissue hyperplasia; nerve markers (NF200 and S100) positive at muscular sites.	In injured spinal segments, S100 was increased and NF-200 was diminished.	This method of NLUTD induction was without the leg’s neurological deficit, easily applicable, low-cost, and was accompanied by minimal surgical preparation and a satisfactory survival rate in comparison with other SCI animal models.
Horst et al., 2013 [42]	SCI	Rat	Female	Two opposite lateral hemisections (T7 and T11)	Awake cystometry: increased number of non-voiding contractions, showing signs of detrusor overactivity; the treatment significantly attenuated bladder dysfunction, but not to basal levels.	PGP 9.5 (general nerve marker) was increased in trained rats and decreased in non-trained rats (reduced detrusor hypertrophy); NF200 afferent fiber innervation was reduced in non-trained animals; the NF200:PGP ratio was significantly lower in trained RATS; non-trained rats showed a trend for low TH density.		A multisystem neuroprosthetic training program counteracts the emergence of neurogenic bladder dysfunction and improves bladder function in rats with severe SCI.
Frias et al., 2015 [27]	SCI	Rat	Female	Complete transection at T9	Cystometry under urethane anesthesia: one week after SCI, the animals presented bladder areflexia; four-week SCI, NDO was evident, with increase in the values of frequency, peak pressure, baseline pressure, and amplitude of urinary function; chronic BDNF sequestration during the first week promoted an earlier appearance of NDO; BDNF sequestration starting at 4 weeks almost abolished bladder contractions.		Time-dependent increase in BDNF expression: L5–L6 segments; GAP-43 expression increased after SCI in thin fibers coursing in the superficial laminae of the dorsal horn; continuous BDNF sequestration during spinal shock resulted in intense GAP-43 expression; increase in phosphoJNK in laminae I–II of the experimental groups in which axonal sprouting was more pronounced.	BDNF is involved in the long-term maintenance of NDO arising after SCI, as an important modulator of sensory afferent sprouting, the key mechanism underlying NDO emergence.
Coelho et al., 2016 [43]	SCI	Rat	Female	Complete transection at T8	Cystometry under urethane anesthesia: increased frequency and basal pressure; decreased amplitude of contractions; treatment with botox normalized these parameters to basal conditions.		Onabot/A cleaves SNAP-25 in L5-S1 spinal segments, coursing laminae I and II of the dorsal horns. Increase in CGRP expression at L5-S1 spinal cord (laminae III and IV) and at DRG level; treatment reduced this. Increase in ATF3 (marker of neuronal stress) expression; treatment further increased this.	Botulinum toxin A improves SCI-induced NDO, acting predominantly on bladder sensory fibres. The mechanism of action of Onabot/A includes the cleavage of SNAP-25 in sensory terminals but also impairment of basic cellular machinery in the cell body of sensory neurons.
Doyle et al., 2018 [44]	SCI	Rat	Male	Complete transection at T8		The level of the muscarinic M2 receptor was decreased in the mucosa of SCI bladders.		Detrusor-mucosa interactions are substantially altered in the neurogenic bladder.
Song et al., 2022 [45]	SCI	Rat	Female	Contusion at T10	Awake cystometry: the basic pressure, maximum pressure, the pressure of leakage point, maximum capacity, residual urine volume, and internal bladder pressure were significantly increased in the SCI group compared with non-SCI groups; treatment improved these features.	-Bladder was enlarged, with a rough and thicker bladder wall; -Abnormal mucosa (both epithelium and lamina propria) with a significant inflammatory infiltrate with neutrophils and monocytes, accompanied by microvascular rupture and bleeding. Treatment improved lamina propria inflammation.	Reduction in mRNAs and proteins of TRPA1, TRPV1, P2X2, and P2X3 both in bladder and L6-S1 DRG; PTNS treatment attenuated this.	Posterior tibial nerve stimulation (PTNS) improves SCI-induced functional and histological impairment of the bladder, via the TRP/P2X signaling pathway.
Wada et al., 2017 [46]	SCI	Mouse	Female	Complete transection at the T8–T9 level	Awake cystometry: bladder capacity, post-void residual urine, and the number of non-voiding contractions during storage were larger when the bladder of SCI animals was only squeezed once daily, compared with twice and thrice.	-At 4 weeks after SCI, the bladder weight was reduced in animals who had their bladders more frequently squeezed; -Levels of NGF protein in the bladder mucosa of SCI mice were higher; -Levels of NGF were lower in animals who had their bladders more frequently squeezed.	The expression of P2X2, P2X3, TRPA1, and TRPV1 mRNA was increased in SCI mice (DRG), when compared to spinal intact mice.	The post-injury bladder management with an increased number of daily bladder emptying improves the storage and voiding LUTD after SCI, associated with the decrease in bladder NGF and reductions in C-fiber afferent marker receptors in bladder afferent pathways.
Sartori et al., 2022 [47]	SCI	Rat	Female	Incomplete transection at the T8–T9 level	Awake cystometry: Increased maximum intravesical pressure, voiding time, post-void residual volume, and the number of non-voiding contractions. Reduced maximum flow rate, voided volume, and voiding efficiency.		Reduction in the density of 5-HT-positive fibers in both lamina X and ventral horn. 5-HT density increased over time, but remains severely affected up to 4 weeks after SCI; -Decrease in CRF-positive fiber density in the intermedio-lateral column (and lamina X), but partially at 4 weeks; -Increase in CGRP density only 2–3 weeks after SCI; -Decrease in the glutamatergic neurons (VGLUT2 mRNA-positive) in the laminae I, II and III of the dorsal horn, but not in laminae IV–V and X; -Decrease in GABAergic cells (GAD2 mRNA-positive) in the laminae I, II, III, IV and V.	Detrusor overactivity is possibly influenced by the sprouting of afferent fibers of type C in the dorsal horn responding to bladder distension, while DSD might be driven by decreased bulbospinal input to and a reduced number of inhibitory GABAergic interneurons in the lumbosacral cord.
Elkelini et al., 2012 [48]	SCI	Rat	Female	Complete transection at T4	Awake cystometry: increased number of non-voiding contractions, basal pressure, and increase in maximum voiding pressure; intravesical onabotA reduced the number of uninhibited contractions and lowered the maximum pressure, but had no effect on resting pressure.	Increase in NGF bladder concentration; onabotA treatment significantly reduced NGF levels.	(3 weeks after SCT) Increase in NGF concentration in the dorsal root ganglia (DRG) of the T4 root; onabotA treatment decreased NGF concentration.	Intravesical onabotulinumtoxinA blocks autonomic dysreflexia in rats with T4-SCT, due to a decrease in NGF concentrations at the bladder and dorsal root ganglia T4, which suggests an afferent pathway modulation by intravesical onabotA treatment.
Shimizu et al., 2017 [49]	SCI	Mouse	Female	Complete transection of T8–T9			-Increase in the number of CGRP and TRPV1 promoter vector-labeled cells in L1 and L6 DRG; the median cell size of CGRP neurons was increased, while TRPV1 was decreased; -Decrease in the number of NF200 promoter vector-labeled cells in L6 DRG; the median cell size of the NF200 neurons was larger.	SCI induces morphological changes in bladder afferent pathways, especially in the C-fiber cell population, along with upregulation of CGRP and TRPV1 expression in bladder afferent neurons.
Takahashi et al., 2013 [50]	SCI	Rat	Female	Complete transection at T9–T10			The ratio of Kv1.4 α-subunit staining density in bladder afferent and unlabeled DRG neurons was lower in spinal transected animals.	The excitability of C-fiber bladder afferent neurons is increased in association with reduction in KA current density and Kv1.4 α-subunit expression in SCI rats.
Munoz et al., 2017 [51]	SCI	Rat	Female	Bilateral dorsal lesion T8–T9	Cystometry under urethane anesthesia: SCI increased duration of intraluminal pressure, high-frequency oscillations, and non-voiding contractions’ frequency; These parameters were improved by P2X7R antagonist treatment.	-Increased expression of beta-actin marker; -Increased levels of urothelial P2X3 receptors; treatment with P2X7R antagonist attenuated both findings.	-Activation and infiltration of microglia in T7/T8 dorsal horn areas in non-BBG treated SCI groups. -The density of CD11b-positive microglia cells and the percentage of activated microglia were significantly reduced in treated rats.	P2X7R antagonist (BBG) induced a significant reduction in the frequency of non-voiding detrusor contractions, which was correlated with a lower amount of activated microglia.
Sartori et al., 2022 [52]	SCI	Rat	Female	Hemisection at T8–T9	Awake cystometry: transcutaneous tibial nerve stimulation induced fewer episodes of non-voiding contractions, a lower maximum intravesical pressure during the storage phase, a higher voided volume, and a lower post-void residual volume in SCI rats, resulting in a higher voiding efficiency; the beneficial effect in bladder urodynamics disappeared one week after the end of the stimulation period.	The unstimulated sham animals had a bigger and heavier bladder compared with animals that underwent tibial nerve stimulation.	Higher density of CGRP-positive structures in layer I and II of the dorsal horn of L6 and S1 in the stimulated group (not statistically significant).	Application of transcutaneous tibial nerve stimulation in rats early after SCI had a beneficial influence on the development of lower urinary tract dysfunction that typically arises after an incomplete SCI.
Cho et al., 2014 [53]	SCI	Rat	Male	T11 damage using a surgical gouge	Cystomery under zolotyl anesthesia: increase in contraction pressure and the contraction time; oral mucosa stem cells transplantation into the injury area ameliorated these features		The transplantation of oral mucosa stem cells decreased the SCI lesion, once new tissues were increased in the surroundings of the damaged tissues, reduced apoptosis, and increased the spinal cord tissues SMA-α and Ki67 expressions; c-Fos and NGF expression in the neuronal voiding centers in SCI animals were also reduced by the treatment.	Transplantation of oral mucosa stem cells ameliorated the SCI-induced neurogenic bladder symptoms by inhibiting apoptosis and enhancing cell proliferation. As result, SCI-induced neuronal activation in the neuronal voiding centers was suppressed, showing the normalization of voiding function.
Yao et al., 2022 [54]	SCI	Rat	Unspecified	Spinal compression at T13	Cystometry under chloral hydrate: placental mesenchymal stem cell-derived neural cell transplantation increased the maximum bladder capacity and bladder compliance, and decreased the bladder basic pressure and urinary leakage pressure in SCI rats.	Placental mesenchymal stem cell-derived neural cell transplantation improves the ultrastructure of detrusor muscle tissue (reduces the rough endoplasmic reticulum, mitochondrial swelling, and thigh fibrils) and improves the elasticity and compliance of detrusor muscles.	-SCI up-regulates caspase-3 protein expression in the spinal cord; the TUNNEL value after placental mesenchymal stem cell transplantation decreased significantly in SCI rats.	Placental mesenchymal stem cell transplantation increased bladder dysfunction after SCI. Cell transplantation can rapidly differentiate into nerve cells to compensate for the massive apoptosis of cells associated with the lesion.
Elkelini et al., 2012 [55]	SCI	Rat	Female	Complete transection at T10	Awake cystometry: transected rats developed uninhibited contractions, increased resting pressure, increased threshold pressure, and increased maximum voiding pressure; short-term sacral neurostimulation reduced the threshold pressure, but no other urodynamic parameters were significantly affected.		Increase in CGRP content (L6DRG); neurostimulation reduced this,	Sacral neuromodulation (SNM) reduced threshold pressure and CGRP content at L6 DRG, which may explain the modulatory effect on the C-fiber afferents supplying the urinary bladder. Chronic stimulation may be required to produce significant changes in all CMG parameters.
Wang et al., 2012 [56]	SCI	Rabbit	Female	Spinal compression by aneurysm clip at T10	Awake cystometry: 2 weeks after SCI, basal pressure, leak-point pressure, and residual urine volume increased; the detrusor was hyperactive during bladder filling, DSD occurred during voiding; bladder compliance was decreased. Four weeks of accumulated sacral anterior root stimulation of anodal block: intravesical pressure, maximum bladder pressure, maximum detrusor pressure, bladder leak-point pressure, resting pressure, and residual volume decreased, while bladder capacity and voiding volume increased.	-Bladder expression of the M2 receptor, P2X3 receptor, and NGF increased in SCI animals; decreased after 4-week electrical stimulation; -Expression of the M3 receptor and β2-adrenergic receptor decreased following SCI, increasing after 4-week electrical stimulation.		Long-term sacral anterior root stimulation of anodal block in rabbits following SCI could repair urinary function. The recovery neurotransmitter receptor expression and decreased NGF expression could be one of the mechanisms of action.
Liu et al., 2018 [57]	SCI	Rat	Female	Complete transection at S2	Cystometry under urethane anesthesia: typical voiding contractions of the bladder were not observed in SCI rats, they were replaced by several irregular micturition waves with low amplitude.	-Detrusor hypertrophy; -Increase in mesenchyme matter; -Increase in bladder volume; -The mRNA and protein expression levels of four HCN subtypes were decreased, with the HCN1 channel being the most significant; all four HCN subtypes were expressed in single bladder interstitial cells of Cajal-like cells (ICC-LCs); -The protein levels of Trip8b, Nedd4-2, and NRSF were upregulated, while filamin A was downregulated.		Decreased bladder HCN channel expression and function induced by altered regulatory proteins are involved in the pathological process of SCI-induced neurogenic bladder.
Han et al., 2017 [58]	SCI	Rat	Female	T11 damage using a surgical gouge	Cystometry with zolotyl anesthesia: increased basal contraction pressure and contraction time; tamsulosin treatments decreased the basal contraction pressure and time, dose-dependently.		Increased c-Fos, NGF and NADPH-d expression in the vlPAG, PMC, and spinal dorsal horn (L5); tamsulosin treatment suppressed these increases.	Tamsulosin (α1-adrenoceptor antagonist) treatment suppressed NDO symptoms and c-fos and NGF augmentation after SCI, showing that it can be used to attenuate bladder dysfunction following SCI.
Yang et al., 2017 [59]	SCI	Rat	Female	Contusion at T10	Awake cystometry: reduction in inter-contraction interval, voided volume, and voiding efficiency; increased basal pressure, threshold pressure and bladder capacity. Bladder function was improved by treatment with tanshinone IIAh methylprednisolone.	-Increased bladder weight; -Increase in thickness of bladder detrusor; -Vascular alterations, edema, and proliferation of urothelial layers; the umbrella cell layer was disrupted and a marked neutrophil infiltration to the suburothelial tissue as well as blood vessel congestion and dilation was observed; treatment with tanshinone IIA and methylprednisolone reduced these features.	-Decrease in motor neurons in the anterior horn, paired with a reduction in Nissl body conspicuity; -DRGs L6-S1 presented a large number of inflammatory cells; -DRGs L6-S1 neurons cell bodies became hypertrophic and elongated with some of the nuclei shrunken or disappeared. Some Nissl bodies also disappeared or were replaced by vacuoles. All these features were attenuated by Tanshinone IIA and methylprednisolone treatments.	Tanshinone IIA and methylprednisolone improved functional recovery after SCI-induced lower urinary tract dysfunction by remodeling the spinal pathway involved in lower urinary tract control.
Lee at al., 2012 [60]	SCI	Rat	Female	Contusion at T8		-Increased bladder weight; -Increased mRNA expression of GAPDH. In the control group, the most-expressed α1 AR subtype was α1a and in the SCI group α1d, but SCI had lower expression than all the suited receptors (α1a, α1b, α1d).		SCI modulates the α1 AR mRNA subtypes in rat urinary bladder. The relatively increased α1d or decreased α1a AR mRNA expression may be a therapeutic candidate for controlling symptoms of neurogenic bladder after SCI.
Cho et al., 2020 [61]	SCI	Rat	Male	Contusion at T8	Awake cystometry: decreased bladder contraction pressure and contraction time; increased intercontractional interval. TGN administration attenuated these changes.		Increased VEGF, NGF, and BDNF expression in spinal injury site; TGN treatment suppressed the expression.	Treatment with a PTEN inhibitor (TGN) induced functional recovery and resulted in significantly lower expression of VEGF, NGF, and BDNF at injury site.
Cui et al., 2021 [62]	SCI	Rat	Male	Complete transection at T9		-Increase in collagen and reduction in smooth muscle fibers; disorganization of these fibers’ distribution; -The ratio of type I/III collagen in bladder smooth muscle cells was higher than in controls. Treatment with 3-methyladenine improved the overall histological changes.	-Enlargement of the space around the nerve cells in the spinal cord; appearance of blurred nucleolus, swollen cells, and vaculose. After treatment, the number of necrotic nerve cells and vacuoles in the spinal cord tissue was reduced and the degree of inflammatory infiltration was reduced; -Increased LC3-II expression levels; treatment reduced them; -Reduced MBP expression; treatment increased them.	3-methyladenine reduces the loss of MBP and inhibits bladder detrusor dysfunction by inhibiting the autophagy response in bladder detrusor muscle cells. The inhibition of collagen fiber expression in the detrusor promotes the recovery of bladder function.
Shimizu et al., 2021 [63]	SCI	Mouse	Female	Complete transection at T8–T9	Awake cystometry: the number of NVCs was significantly reduced in vibegron-treated SCI mice compared to vehicle-treated control SCI mice.		Increased mRNA levels of TRPV1, TRPA1, ATF3, and iNOS in L6-S1 DRG were increased in SCI mice versus SI mice and decreased after vibegron treatment.	β3-adrenoceptor activation by vibegron improved the SCI-induced storage dysfunction, possibly through the reduction in C-fiber-related receptor expression and inflammation-related markers in DRG.
Gil-Tomee et al., 2019 [64]	PD	Mouse	Both	GM2 synthase knockout (KO) mice	KO mice initially had more void spots that reduced with age. KO mice initially had bladder hyperreflexia and then developed hyporeflexia.	-Increased proNGF protein levels; -Abnormal myelination in bladder nerves.	Loss of TH in the VTA of GM2 KO mice compared to WT mice.	Dopaminergic damage in brain micturition centers impact voiding in association with a loss in mature ganglioside.
McMillan et al., 2014 [65]	MS	Mouse	Male	Experimental autoimmune encephalomyelitis (EAE)	Voiding spot assay: increased number of non-voiding contractions; decreased voided volume; shortened intermicturition intervals; decreased bladder capacity; reduced volume of voided urine.		-Upregulation of GFAP expression (gliosis) and decrease in MBP at the lesion site; -Several proinflammatory cytokines were upregulated in the brains at 1 wk, followed by a gradual recovery to baseline values by 4 wk -In the spinal cord, only IFN-γ levels were upregulated at 1 wk, suggestive of a mild inflammatory reaction; normal levels were observed at 4 wk.	Coronavirus-induced demyelination of the CNS causes the development of a neurogenic bladder that is comparable with neurogenic detrusor overactivity observed in patients with multiple sclerosis.
Altuntas et al., 2012 [66]	MS	Mouse	Female	Experimental autoimmune encephalomyelitis (EAE)		-Increase in collagen; -Decrease in NGF, GDNF, muscarinic receptors, and purinergic receptors; -Increased expression of CTGF and TGF-β3 mRNAs (markers of the fibrosis cascade),		EAE-caused neurological disability in mice and contributes to marked bladder remodeling. Although all three components of detrusor smooth muscle, urothelium, and connective tissue contribute to the increased bladder mass, the role of connective tissue is more prominent and potentially detrimental.
Lee et al., 2019 [67]	MS	Mouse	Male	Coronavirus-induced encephalitis (CIE)	Voiding spot assay: at 10 wks post-inoculation, bladder capacity, the inter-micturition interval, and bladder pressure at voiding in all groups, except for the C-RELAP group, were similar to the respective values in the control group. Mice in the C-RELAP group developed overactive bladder phenotype. This means that the C-RELAP group develop a more severe and long-lasting type of neurogenic bladder overactivity than other groups, providing evidence of some correlation between the type of neurodegenerative changes in the CNS and type of developed voiding dysfunction in CIE mice.	Increased expression of TNF-α, Increased content of IFN-γ, IL-2, TGF-β and TNF-α	Decreased expression of IL-1β and IL-10 in the brain. The C-PRO group was characterized by a decreased expression of IL-1β, IL-6, IL-10, IL-17, and TNF-α. C-RELAP mice had a significantly reduced level of IL-4 in the brain.	Mice with CIE developed three phenotypes of neurologic impairment, mimicking different types of MS.
Jin et al., 2017 [68]	MS	Mouse	Unspecified	Experimental autoimmune encephalomyelitis (EAE)	Voiding spot assay: decrease in urine volume voided per micturition and increased frequency of urination; increased bladder diameter; features reverted with treatment.	-Decreased number of ICCs (c-Kit staining); treatment partially reverted this; -Increased expression of Panx1 and Cx43.		The effect of SCF on the loss of ICCs may offer a theoretical option for treating patients with MS-related voiding dysfunction.
Xue et al., 2013 [69]	MS	Mouse	Female	Experimental autoimmune encephalomyelitis (EAE)	-EP3 and EP4 agonists increased micturition frequency and decreased void weight in EAE mice, compared with control mice treated with vehicles.	-The concentration of PGE2 level in the bladder increased as the EAE (severity) score increased; -Bladder weight to total body weight ratio was higher; -The expression of EP3 and EP4 receptors increased as EAE severity score increased, but no change in expression of EP1 and EP2 receptors was verified.		EAE-induced upregulation of EP3 and EP4 receptors in the bladder was accompanied by bladder dysfunction. However, EAE had no significant effect on EP1 and EP2 receptors.
Liang et al., 2016 [70]	Cerebral ischemia	Rat	Female	Middle cerebral artery occlusion (MCAO)	-Increased peak voiding pressure and residual volume at 1 day, 3 days, and 7 days following ischemia induction, which declined after pre- and postischemic CD34+ cells treatment; -Decreased voided volumes and intercontraction intervals decreased after ischemia induction, but increased after pre- and postischemic CD34+ cell treatment at 3 days and 7 days.	NGF, M2, and M3 mRNA expression and immunoreactivity (except for M2) decreased after MCAO, but increased after preischemic CD34+ cell treatment.		Bladder dysfunction caused by MCAO may have a normal micturition restored by treatment with human umbilical cord blood-derived CD34+ cells. This urinary function improvement may be related to the expression of bladder NGF, M2, and M3.
Campeau et al., 2014 [71]	PD	Rat	Female	Injection of 6-hydroxydopamine in the right medial forebrain bundle	Awake cystometry: increased bladder capacity, post-void volume, and threshold pressure; decreased after treatment with rBMSCs; urodynamic effects of the 6-OHDA lesion up to 42 days after injection.		-GFAP expression was present around rBMSC and ErBMSC graft sites, unlike the saline injection site, suggesting activated astrocytes around the graft sites; -There was IBA-1 expression at the injection site in all groups, but it vanished by the fourth week. Microglia infiltration was present around injected rBMSCs and ErBMSCs.	Transplantation of rBMSCs alone improved urodynamic pressure at 42 days after treatment more markedly than ErBMSCs. This improvement in rBMSC rats was associated with a higher number of TH-positive neurons in the treated SNpc, suggesting that functional improvement may require a juxtacrine effect.
Cho et al., 2015 [72]	Intracerebral hemorrhage (ICH)	Rat	Female	Induction of ICH in the hippocampal CA1 region was performed using a stereotaxic frame and collagenase	Cystometry under zolotyl anesthesia: bladder contraction pressure and time were significantly increased and the voiding pressure and time decreased by the induction of ICH, as compared with the control treatment.		-c-Fos expression levels in the neuronal voiding centers (medial preoptic area, ventrolateral gray, pontine micturition center, and SC L4-L5) were increased; -NGF expression levels in the neuronal voiding centers were increased.	ICH-induced NLUTD rat model may be a more appropriate method to analyze NLUTD in stroke patients than a cerebral infarction model.
Pritchard et al., 2017 [73]	PD	Marmoset	Both	MPTP injection			-Reduction in tyrosine hydroxylase expression in the substantia nigra.	The increased neurogenically mediated contractions where no extrinsic innervation exists might be due to long-term adaptive changes locally as a result of the loss of the nigrostriatal output.
Shen et al., 2013 [74]	Meningomyelocele	Rat	Fetuses from female rats	Gavage feeding of retinoic acid at embryonic day 10 (E10)		Decrease in β-III-Ttubulin- content at E16, E18, and E20.	-Increase in GFAP expression (in the dorsal region of the spinal cord); -Decrease in VAChT expression in the dorsal lateral nucleus of the spinal cord at all fetal ages.	Smooth muscle of the bladder in fetal rats with myelomeningocele is morphologically normal, while the innervation of the smooth muscle of the bladder is markedly decreased centrally and peripherally. Astrocytosis appears in a later embryonic stage, which could be related to nerve repair in the spinal cord.
Tekin et al., 2016 [75]	Myelomeningocele	Rat	Fetuses from pregnant female rats	Gavage feeding of retinoic acid at embryonic day 10 (E10)			-The interstitial cells of Cajal (ICC) score of the MMC group is decreased.	The density of the ICC in the urinary bladder decreased in the neurogenic bladder developed in MMC.
Liu et al., 2022 [76]	Myelomeningocele	Rat	Fetuses from pregnant rats	Gavage feeding of retinoic acid at embryonic day 10 (E10)		-Inhibition of bladder cells proliferation, due to increased apoptosis in late embryonic stage (increased cleaved caspase 3); -Increase in α-SMA mRNA; -NeuN protein expression increased with time, with no significant difference between the MMC and CRL groups from E16 to E18; however, the expression of NeuN protein was significantly lower in the MMC group than in the CRL group from E20 to E22.		Bladder dysfunction in myelomeningocele fetal rats is related to the inhibition of proliferation, promotion of apoptosis, and reduction in bladder nerve and smooth muscle-related protein synthesis.

**Table 2:** Expression of molecular markers in the bladder wall after NDO induction according to NDO model and tissue layer. Every molecule with a statistically significant expression variation (*p* < 0.05) in the included papers is present in this table. Molecules are split into categories and ordered alphabetically. BDNF: brain-derived neurotrophic factor, GDNF: glial cell line-derived neurotrophic factor, NGF: nerve growth factor, IFN-γ: interferon-gamma, IL: interleukin, TNF-α: tumor necrosis factor alpha, GAPDH: glyceraldehyde 3-phosphate dehydrogenase, EP: prostaglandin E_2_ receptor, pTrkA: phosphorylated tropomyosin receptor kinase A, HCN channels: hyperpolarization-activated cyclic nucleotide-gated channel, Panx 1: pannexin 1, TRPA-1: transient receptor potential ankyrin-1, TRPM-4: transient receptor potential melastin-4, TRPV-1: transient receptor potential vanilloid-1, NF200: neurofilament 200, NPY: neuropeptide Y, PGP 9.5: protein gene product 9.5, SYP: synaptophysin, CTGF: connective tissue growth factor, FGF: fibroblast growth factor, HIF-1α: hypoxia-inducible factor 1-alpha, TGF: transforming growth factor, VEGF-α: vascular endothelial growth factor-alpha, Cx 43: connexin 43, Nedd4-2: neural precursor-expressed developmentally down-regulated protein 4-2, NRSF: neuron-restrictive silencer factor, pAkt: phosphorylated Ak strain transforming, Trip8b: tetratricopeptide-repeat containing Rab8b-interacting protein. ↑: expression increase, ↓: expression decrease, †: expression variation detected through protein analysis, ‡: expression variation detected through RNA analysis, CI: cerebral infarction, MMC: myelomeningocele, MS: multiple sclerosis, PD: Parkinson’s disease, SCI ^a^: acute spinal cord injury (defined here by ≤14 days from NDO induction), SCI ^c^: chronic spinal cord injury (defined here by >14 days from NDO induction), M: mucosa, D: detrusor (either detrusor smooth muscle or detrusor stroma), W: whole bladder, E20–22: expression variation only detected on embryonic day 20–22, *: return to basal between 1 and 2 months after NDO induction.

Molecular Factors	NDO Model	Tissue Layer	Bladder Expression after NDO Induction	Treatments That Reverted or Attenuated NDO-Related Expression Change	
**Neurotrophic factors**					
BDNF † [35]	SCI ^a,c^	M	↑		
NGF †‡ [29,46,48,56]	SCI ^c^	W †‡	↑	siRNA Intravesical onabotulinumtoxinA	
		M †	↑	Increased number of daily bladder emptyings	
		D †	↑	Long-term sacral anterior root stimulation	
**Apoptosis-related factors**					
GAPDH ‡ [60]	SCI ^c^	W	↑		
**Receptors**					
M2 receptor †‡ [44,56]	SCI ^c^	M †‡	↓		
		D †	↑	Long-term sacral anterior root stimulation (detrusor stroma)	
M3 receptor †‡ [33,44,56]	SCI ^c^	M ‡	↓		
		D †	↓	Long-term sacral anterior root stimulation (detrusor stroma)	
		W ‡	↓	M3 RNAi lentivirus	
pTrkA † [29]	SCI ^c^	W	↑		
P2X2 †‡ [45]	SCI ^c^	W	↓	Posterior tibial nerve stimulation (PTNS)	
P2X3 †‡ [45,51,56]	SCI ^c^	W †‡	↓	Posterior tibial nerve stimulation (PTNS)	
		M†	↑	BBG (P2X7R antagonist) Long-term sacral anterior root stimulation	
α1a, α1b, α1d -adrenergic receptors ‡ [60]	SCI ^c^	W	↓		
β2-adrenergic receptors † [56]	SCI ^c^	M	↓	Long-term sacral anterior root stimulation	
**Ionic channel proteins**					
HCN channel proteins †‡ [57]	SCI ^c^	W	↓		
TRPA-1 †‡ [45]	SCI ^c^	W	↓	Posterior tibial nerve stimulation (PTNS)	
TRPM-4 † [30]	SCI ^a^	M, D	↑*	9-phenanthrol (TRPM-4 antagonist)	
TRPV-1 †‡ [26,32,45]	SCI ^c^	W	↑†^c^ ↓†‡^c^	Inosine (decreases) Neurotoxin RTX (decreases) Posterior tibial nerve stimulation (PTNS) (increases)	
**Neuronal markers**					
NF200 † [32,41,42]	SCI ^c^	W	↓	Inosine Multisystem neuroprosthetic training program	
		D	↓		
NPY † [42]	SCI ^c^	W	↓	Multisystem neuroprosthetic training program	
PGP 9.5 † [42]	SCI ^c^	W	↓	Multisystem neuroprosthetic training program	
SYP † [32]	SCI ^c^	W	↓	Inosine	
β-III-tubulin † [36]	SCI ^c^	W	↑		
**Ischemia- and fibrosis-related molecules**					
FGF-2 ‡ [31]	SCI ^c^	W	↑		
FGF-β ‡ [39]	SCI ^c^	W	↑	Oxybutynin (anti-cholinergic agent)	
HIF-1α ‡ [31,39]	SCI ^c^	W	↑	Vibegron and mirabegron (β3-adrenoceptor agonists) Oxybutynin (anti-cholinergic agent)	
TGF-β1 †‡ [31,34,39]	SCI ^c^	W	↑	Vibegron and mirabegron (β3-adrenoceptor agonists) Oxybutynin (anti-cholinergic agent) BoNTA injection	
VEGF-α ‡ [31]	SCI ^c^	W	↑		
**Other molecules**					
Filamin A † [57]	SCI ^c^	W	↓		
Nedd4-2 † [57]	SCI ^c^	W	↑		
NRSF † [57]	SCI ^c^	W	↑		
pAKT † [29]	SCI ^c^	W	↑		
Trip8b † [57]	SCI ^c^	W	↑		
**Molecular Factors**	**NDO Model**	**Site**	**Bladder Expression after NDO Induction**	**Treatments That Reverted or Attenuated NDO-Related Expression Change**	
**Neurotrophic factors**					
GDNF ‡ [66]	MS	W	↓		
NGF ‡ [66]	MS	W	↓		
**Inflammatory mediators**					
IFN-γ † [67]	MS	W	↑		
IL-2 † [67]	MS	W	↑		
TNF-α † [67]	MS	W	↑		
**Receptors**					
c-kit † [68]	MS	W	↓	Stem cell factor cytokine injection	
EP-3 and EP-4 † [69]	MS	W	↑		
M3 receptor ‡ [66]	MS	W	↓		
P2X1 ‡ [66]	MS	W	↓		
**Ionic channel proteins**					
Panx 1 † [68]	MS	W	↑	Stem cell factor cytokine injection	
**Ischemia- and fibrosis-related molecules**					
CTGF ‡ [66]	MS	W	↑		
TGF-β1 † [67]	MS	W	↑		
TGF-β3 ‡ [66]	MS	W	↑		
**Other molecules**					
Cx 43 † [68]	MS	W	↑	Stem cell factor cytokine injection	
**Molecular Factors**	**NDO Model**	**Site**	**Bladder Expression after NDO Induction**	**Treatments That Reverted or Attenuated NDO-Related Expression Change**	
**Neurotrophic factors**					
NGF †‡ [70]	CI	W	↓	Treatment with human umbilical cord blood-derived CD34+ cells	
**Receptors**					
M2 receptor †‡ [70]	CI	W	↓‡ ↑†	Treatment with human umbilical cord blood-derived CD34+ cells	
M3 receptor †‡ [70]	CI	W	↓	Treatment with human umbilical cord blood-derived CD34+ cells	
**Molecular Factors**	**NDO Model**	**Site**	**Bladder Expression after NDO Induction**	**Treatments That Reverted or Attenuated NDO-Related Expression Change**	
**Neurotrophic factors**					
Pro-NGF † [64]	PD	W	↑		
**Molecular Factors**	**NDO Model**	**Site**	**Bladder Expression after NDO Induction**	**Treatments That Reverted or Attenuated NDO-Related Expression Change**	
**Apoptosis-related factors**					
Caspase-3 † [76]	MMC	M, D	↑		
**Neuronal markers**					
NeuN † [76]	MMC	W (E20–22)	↓		
β-III-tubulin † [74]	MMC	D	↓		

**Table 3:** Expression of molecular markers in the nervous system (NS) after NDO induction according to NDO model and NS site. Every molecule with a statistically significant expression variation (*p* < 0.05) in the included papers is present in this table. Molecules are split into categories and ordered alphabetically. BDNF: brain-derived neurotrophic factor, GAP-43: growth-associated protein 43, NGF: nerve growth factor, IFN-γ: interferon-gamma, IL: interleukin, PGE2: prostaglandin E_2_, TNF-α: tumor necrosis factor alpha, CSPG-R: chondroitin sulfate proteoglycans receptors, EP: prostaglandin E_2_ receptor, MAI-R: myelin-associated inhibitors receptors, ASIC: acid-sensing ion channel, Kv1.4 α-subunit: voltage-activated Shaker-related potassium channel 1.4 alpha-subunit, TRPA-1: transient receptor potential ankyrin-1, TRPV-1: transient receptor potential vanilloid-1, 5-HT: 5-hydrxytryptamine, CGRP: calcitonin gene-related peptide, CRF: corticotropic-releasing factor, GAD2: glutamic acid decarboxylase, NF200: neurofilament 200, TH: tyrosine hydroxylase, VAChT: vesicular acetylcholine transporter, VGLUT-2: vesicular glutamate transporter 2, VEGF-α: vascular endothelial growth factor-alpha, MAG: myelin-associated glycoprotein, MBP: myelin basic protein, OMgp: oligodendrocyte-myelin glycoprotein, RGMa: repulsive guidance molecule A, ATF-3: cyclic AMP-dependent transcription factor-3, GFAP: glial fibrillary acidic protein, iNOS: inducible nitric oxide synthase, LC3-II: microtubule-associated protein 1A/1B-light chain 3-phosphatidylethanolamine conjugate, NADPH-d: dihydronicotinamide adenine dinucleotide phosphate diaphorase, pJNK: phosphorylated c-Jun N-terminal kinases. ↑: expression increase, ↓: expression decrease, †: expression variation detected through protein analysis, ‡: expression variation detected through RNA analysis, ^•^: expression variation detected through number of cells expressing the studied protein. CI: cerebral ischemia, DRG: dorsal root ganglia, ICH: intracerebral hemorrhage, MMC: myelomeningocele, MS: multiple sclerosis, PD: Parkinson’s disease, SC: spinal cord, SCI ^a^: scute spinal cord injury (defined here by ≤14 days from NDO induction), SCI ^c^: chronic spinal cord injury (defined here by >14 days from NDO induction), only E20–22: expression variation only detected in embryonic days 20 to 22, *: return to basal between 1–2 months after NDO induction, 1 w: 1 week after NDO induction, 10 w: 10 weeks after NDO induction.

Molecular Factors	NDO Model	Location within the Nervous System	Expression after NDO Induction	Treatments That Reverted or Attenuated NDO-Related Expression Change
**Neurotrophic factors**				
BDNF † [27,61]	SCI ^a,c^	Lumbar spinal cord ^a,c^	↑	
		Spinal injury level ^c^	↑	Treatment with a PTEN inhibitor (TGN)
NGF †^•^ [48,53,57,61]	SCI ^c^	T4 DRG †	↑	
		Neuronal voiding centers ^•^	↑	Transplantation of oral mucosa stem cells Tamsolusin
		Spinal injury site †	↑	Treatment with a PTEN inhibitor (TGN)
**Inflammatory mediators**				
PGE2 † [38]	SCI ^c^	Lumbosacral spinal cord	↑	
**Apoptosis-related factors**				
Caspase-3 † [54]	SCI ^c^	Spinal injury level	↑	Placental mesenchymal stem cell transplantation
**Receptors**				
CSPG-R ‡ [28]	SCI ^c^	L5-S1 DRG	↓	
EP-1 ‡ [38]	SCI ^c^	Lumbosacral spinal cord	↑	
MAI-R ‡ [28]	SCI ^c^	L5-S1 DRG	↓	
P2X2 †‡ [45,46]	SCI ^c^	L6-S1 DRG	↑ ‡ ↓ †‡	Increased number of daily bladder emptyings (decreases) Posterior tibial nerve stimulation (PTNS) (increases)
P2X3 †‡ [45,46]	SCI ^c^	L6-S1 DRG	↑ ^c^ ‡ ↓ ^c^ †‡	Increased number of daily bladder emptyings (decreases) Posterior tibial nerve stimulation (PTNS) (increases)
**Ionic channel proteins**				
ASIC1 ‡ [35]	SCI ^a,c^	L6-S1 DRG	↑	
ASIC2 ‡ [35]	SCI ^a,c^	L6-S1 DRG	↑ *	
ASIC3 ‡ [35]	SCI ^c^	L6-S1 DRG	↑ *	
Kv1.4 α-subunit ‡ [50]	SCI ^c^	L6 DRG bladder afferent neurons	↓	
Piezo2 ‡ [35]	SCI ^c^	L6-S1 DRG	↑	
TRPA-1 †‡ [45,46,63]	SCI ^c^	L6-S1 DRGs	↑ ^c^ ‡ ↓ ^c^ †‡	Increased number of daily bladder emptyings (decreases) Vibegron (decreases) Posterior tibial nerve stimulation (PTNS) (increases)
TRPV-1 †‡^•^ [35,45,46,49,63]	SCI ^a,c^	L1, L6-S1 DRG †‡^• a,c^	↑ ^a,c^ ‡^•^ ↓ ^c^ †‡	Increased number of daily bladder emptyings (decreases) Vibegron (decreases) Posterior tibial nerve stimulation (PTNS) (increases)
		L6 DRG bladder afferent neurons ^c^ ‡	↑	
**Neuronal markers**				
5-HT † [47]	SCI ^a,c^	Lumbosacral spinal cord (lamina X and ventral horn)	↓	
CGRP †‡^•^ [26,43,47,49]	SCI ^c^	Lumbosacral spinal cord (Dorsal horn) †	↑	
		L1 and L6 DRG ^•^	↑	Sacral neuromodulation
		L6 DRG bladder afferent neurons ‡	↑	
CRF † [40,47]	SCI ^a,c^	Lumbosacral spinal cord (lamina X and intermediolateral column)	↓	Anti-Nogo-A antibody
GAD2 †‡ [40,47]	SCI ^c^	Lumbosacral spinal cord	↓	Anti-Nogo-A antibody
NF200 ^•^ [49]	SCI ^c^	L6 DRG	↓	
VGLUT-2 ‡ [47]	SCI ^c^	Lumbosacral spinal cord (laminae I-III)	↓	
**Ischemia and fibrosis-related molecules**				
Neurocan † [28]	SCI ^a^	Lumbosacral spinal cord	↑ *	
Phosphacan † [28]	SCI ^a^	Lumbosacral spinal cord	↑ *	
VEGF-α † [61]	SCI ^c^	Spinal injury level	↑	Treatment with a PTEN inhibitor (TGN)
**Myelin-associated proteins**				
MAG † [28]	SCI ^a^	Lumbosacral spinal cord	↑ *	
MBP † [62]	SCI ^a,c^	Undefined (long spinal cord segment)	↓	3-methyladenine
Nogo-A † [28]	SCI ^a^	Lumbosacral spinal cord	↑ *	
OMgp † [28]	SCI ^a^	Lumbosacral spinal cord	↑ *	
RGMa † [28]	SCI ^a^	Lumbosacral spinal cord	↑ *	
**Other molecules**				
ATF3 ‡ [63]	SCI ^c^	L6-S1 DRG	↑	Vibegron
c-Fos ^•^ [53,57,58]	SCI ^c^	Neuronal voiding centers	↑	Transplantation of oral mucosa stem cells Tamsolusin
		Low lumbar spinal cord (dorsal horn)	↑	Tamsolusin
GAP-43 † [26,27]	SCI ^a,c^	Lumbosacral spinal cord ^a,c^	↑	
		L5-S1 DRG ^a^	↑ *	
GFAP † [37]	SCI ^c^	Spinal injury level	↑	
iNOS ‡ [63]	SCI ^c^	L6-S1 DRG	↑	Vibegron
LC3-II † [62]	SCI ^a,c^	Undefined (long spinal cord segment)	↑	3-methyladenine
NADPH-d ^•^ [58]	SCI ^c^	Low lumbar spinal cord (dorsal horn)	↑	Tamsolusin
		Neuronal voiding centers	↑	
pJNK † [27]	SCI ^c^	Lumbar spinal cord	↑	
S100 † [41]	SCI ^c^	Spinal injury level	↑	
**Molecular Factors**	**NDO Model**	**Location within the Nervous System**	**Expression after NDO Induction**	**Treatments That Reverted or Attenuated NDO-Related Expression Change**
**Inflammatory mediators**				
IFN-γ † [65]	MS	Brain	↑ (1 w) *	
		Spinal cord	↑ (1 w) *	
IL-1β † [67]	MS	Brain	↓ (10 w)	
IL-4 † [67]	MS	Brain	↓ (10 w)	
IL-5 † [65]	MS	Brain	↑ (1 w) *	
IL-6 † [65,67]	MS	Brain	↑ (1 w) * ↓ (10 w)	
IL-10 † [67]	MS	Brain	↓ (10 w)	
IL-17 † [65,67]	MS	Brain	↑ (1 w) * ↓ (10 w)	
TNF-α † [65,67]	MS	Brain	↑ (1 w) * ↓ (10 w)	
**Myelin-associated proteins**				
MBP † [65]	MS	Spinal lesion sites	↓	
**Other molecules**				
GFAP † [65]	MS	Spinal lesion sites	↑	
**Molecular Factors**	**NDO Model**	**Location within the Nervous System**	**Expression after NDO Induction**	**Treatments That Reverted or Attenuated NDO-Related Expression Change**
**Neurotrophic factors**				
NGF ^•^ [72]	ICH	Neuronal voiding centers	↑	
**Neuronal markers**				
TH ^•^ [64,73]	PD	Substancia nigra	↓	
		Ventral tegmental area	↓	
**Molecular Factors**	**NDO Model**	**Location within the Nervous System**	**Expression after NDO Induction**	**Treatments That Reverted or Attenuated NDO-Related Expression Change**
**Neuronal markers**				
VAChT † [74]	MMC	Dorsal lateral nucleus of the spinal cord	↓	
**Other molecules**				
GFAP † [74]	MMC	Lumbosacral spinal cord (only E20)	↑	

## Data Availability

Not applicable.
